# Geochemical influences and mercury methylation of a dental wastewater microbiome

**DOI:** 10.1038/srep12872

**Published:** 2015-08-14

**Authors:** Asha Rani, Karl J. Rockne, James Drummond, Muntasar Al-Hinai, Ravi Ranjan

**Affiliations:** 1 Department of Civil and Materials Engineering, University of Illinois, Chicago, IL, USA; 2 Department of Bioengineering, University of Illinois, Chicago, IL, USA; 3 Department of Orthodontics, University of Illinois, Chicago, IL, USA; 4 Department of Medicine, University of Illinois, Chicago, IL, USA

## Abstract

The microbiome of dental clinic wastewater and its impact on mercury methylation remains largely unknown. Waste generated during dental procedures enters the sewer system and contributes a significant fraction of the total mercury (tHg) and methyl mercury (MeHg) load to wastewater treatment facilities. Investigating the influence of geochemical factors and microbiome structure is a critical step linking the methylating microorganisms in dental wastewater (DWW) ecosystems. DWW samples from a dental clinic were collected over eight weeks and analyzed for geochemical parameters, tHg, MeHg and bacterio-toxic heavy metals. We employed bacterial fingerprinting and pyrosequencing for microbiome analysis. High concentrations of tHg, MeHg and heavy metals were detected in DWW. The microbiome was dominated by Proteobacteria, Actinobacteria, Bacteroidetes, Chloroflexi and many unclassified bacteria. Significant correlations were found between the bacterial community, Hg levels and geochemical factors including pH and the predicted total amount (not fraction) of neutral Hg-sulfide species. The most prevalent known methylators included *Desulfobulbus propionicus, Desulfovibrio desulfuricans, Desulfovibrio magneticus* and *Geobacter sulfurreducens*. This study is the first to investigate the impact of high loads of Hg, MeHg and other heavy metals on the dental clinic wastewater microbiome, and illuminates the role of many known and unknown sulfate-reducing bacteria in Hg methylation.

Methyl mercury (MeHg) is a potent neurotoxin that causes significant risk to humans^1^,^2^,^3^ and other top predators through the process of bio-magnifications^4^,^5^. Atmospheric emissions of Hg can be transported over long distances and impact surface waters through deposition and subsequent transformation to the methyl form^6^,^7^. A comparatively less investigated pathway of MeHg input to surface waters is *via* direct dental wastewater (DWW) discharge. Waste material generated from dental offices during dental procedures enters the sewer system and represent a significant fraction of Hg load to wastewater treatment facilities^8^,^9^,^10^,^11^,^12^,^13^. Approximately 50% of mercury entering municipal wastewater treatment plants (WWTPs) comes from dental amalgam waste from discharges totaling 3.7 tons per year in the USA^14^.

Mercury methylation is a natural process that converts Hg (II) to the bio-accumulative toxin MeHg, thought to be primarily mediated by sulfate-reducing and iron-reducing bacteria (SRB and FeRB respectively)^15^,^16^,^17^,^18^,^19^. However, not all SRB and FeRB strains are able to produce MeHg, and to date only few methylating bacteria have been positively identified and sequenced^20^,^21^,^22^. Most SRB that have been confirmed to methylate Hg are in the orders *Desulfovibrionales* and *Desulfobacterales*^23^,^24^,^25^,^26^,^27^. In addition, several species of *Geobacter* and *Desulfuromonas palmitatis* SDBY1 produce MeHg^18^,^19^,^28^. Mercury methylation outside the class Deltaproteobacteria was thought to be limited to Gammaproteobacteria and Firmicutes^27^,^29^, but recent studies have shown Hg methylation among unexpected strains *Pleomorphomonas* sp., unidentified Deltaproteobacteria and *Klebsiella* sp., isolated from the Amazon River^30^. Relatively, little was known about why some strains can methylate Hg while others cannot^24^,^25^,^31^,^32^. Parks *et al.*, identified a specific gene cluster (*hgcAB*) linked to Hg methylation in confirmed methylating bacteria sequenced to date, but absent in non-methylators^33^. These findings were further tested and confirm that the presence of *hgcAB* predicts Hg methylation capability in a number of species other than SRB and FeRB^34^. A recent study using site-directed mutagenesis of the *hgc*AB gene cluster has revealed important amino acid residues required for mercury methylation^35^.

In our previous work, we have shown high levels of Hg and MeHg in DWW collected from dental clinics^12^,^13^. These studies revealed that some Hg methylation was abiotic using dark killed controls, although the first order methylation rate was 2–3 times lower compared to live cultures. The dark environment of the DWW system would preclude any light-mediated abiotic methylation, so the only known abiotic methylation process would be DOC-mediated in DWW^36^. A highly statistically significant correlation was observed between MeHg levels and Desulfobacteraceae and Desulfovibrionaceae DNA in the DWW. Heavy metals and particularly Hg are known to represent a major stress on the microbial community. We hypothesize that the environmental conditions in DWW exert selective pressure on the microbial community composition that favors the dominance of bacteria capable of Hg methylation. The presence of other metals in dental amalgam such as silver suggests that adaptation to metal stress is highly important for DWW community survival, and thus can be a major factor in shaping the DWW microbiome.

The overall objective of the present study is a detailed assessment of the DWW microbiome impacted with high levels of tHg, MeHg and potentially antibacterial heavy metals like Ag, Zn and Cu. A central aim is also to identify the role microorganisms play in DWW samples with and without high levels of MeHg, in particular focusing on the role known Hg methylators like SRB may play in Hg methylation. We applied community fingerprinting of bacteria (ARISA) and massively parallel 16S rRNA gene tag sequencing to increase the resolution of microbial diversity assessments in a 14 sample subset of the entire DWW dataset focusing on those samples with the highest or lowest levels of MeHg. The higher resolution provides insight into the presence or absence of bacterial species which are known to play a key role in Hg methylation in DWW. We also explore Hg methylation within the class Deltaproteobacteria identified in DWW and known Hg methylating SRB using 16S rRNA gene based comparative phylogeny.

## Results

### Description of the DWW sample geochemistry

**Figure 1:**
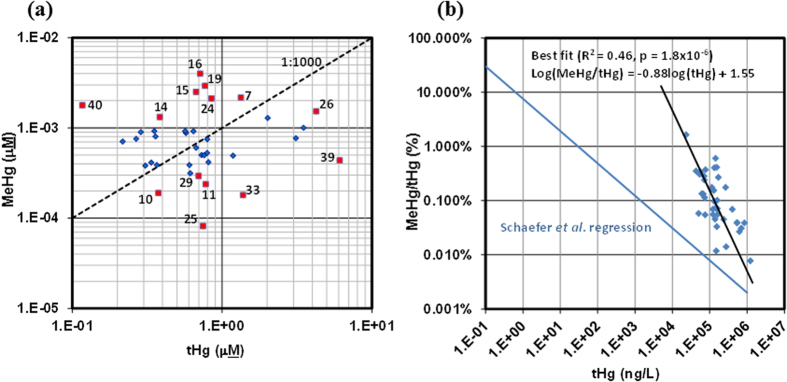
Correlation of MeHg to tHg in DWW. (**a**) Shown are log-transformed tHg and MeHg (μM) concentrations. Fourteen DWW samples with the highest and lowest MeHg/tHg ratios were selected for 16S rRNA gene pyrosequencing (red squares with sample number). Dashed line represents a MeHg/tHg ratio of 1:1000. (**b**) The comparison of the regression of MeHg/tHg versus tHg (in Schaefer *et al.*, 2004)^37^ illustrates the high levels of tHg observed in the present study.

Descriptive statistics for geochemical parameters in the full DWW dataset are compiled in Supplementary Table S1. The results demonstrate that most geochemical parameters were not normally distributed, as shown by both the Shapiro-Wilk and Anderson-Darling tests. In general, Cu, Zn, Hg and MeHg varied by the greatest amount, by up to two orders of magnitude. High levels of these metals were observed in the DWW, with mean ± SEM (standard error of the mean) values of 376 ± 43 μM, 170 ± 19 μM, 21 ± 1.2 μM, 1.1 ± 0.2 μM, and 9.2 × 10^−4^ ± 5.7 × 10^−4^ μM for Cu, Zn, Ag, tHg and MeHg, respectively. MeHg levels in DWW were highly variable ranging from 4.0 × 10^−3^ to 8.2 × 10^−5^ μM. Total Hg in the DWW was much less variable than MeHg, ranging from 0.12–6.1 μM. The highest MeHg concentration detected (4.0 × 10^−3^ μM) was in DWW16 and the lowest (8.2 × 10^−5^ μM) in DWW25 (Fig. 1a). MeHg to tHg ratios (MeHg/tHg) were also highly variable (Fig. 1a,b) spanning two orders of magnitude from 0.01–1.5%. MeHg/tHg versus tHg followed a log-log trend with a slope of −0.88±0.16 (R^2^ = 0.46, *P* = 1.8 × 10^−6^). In comparison, the regression of MeHg/tHg versus tHg in Schaefer *et al.*, 2004^37^, would suggest ratios of approximately 0.01% for the high levels of tHg observed in the present study; at the lowest end of what we observed (Fig. 1b). Based upon these ratios of MeHg to tHg, we observed that a cluster of samples had very high levels of MeHg while a second cluster had lower ratios (Fig. 1a). These 14 samples were the focus of more detailed analysis and the geochemical parameter data for this sample subset is summarized in Table 1.

**Figure 2:**
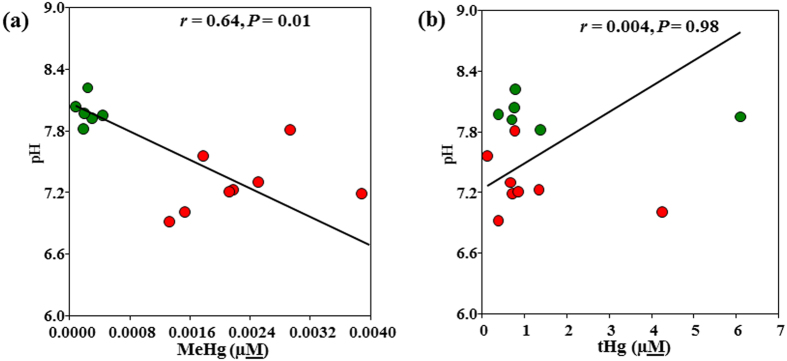
The correlations between pH and MeHg (**a**), pH and tHg (**b**). Red and green dots represent high and low MeHg samples, respectively. Linear regressions were used to test Pearson’s correlation between DWW samples with high and low mercury levels and pH.

**Figure 3:**
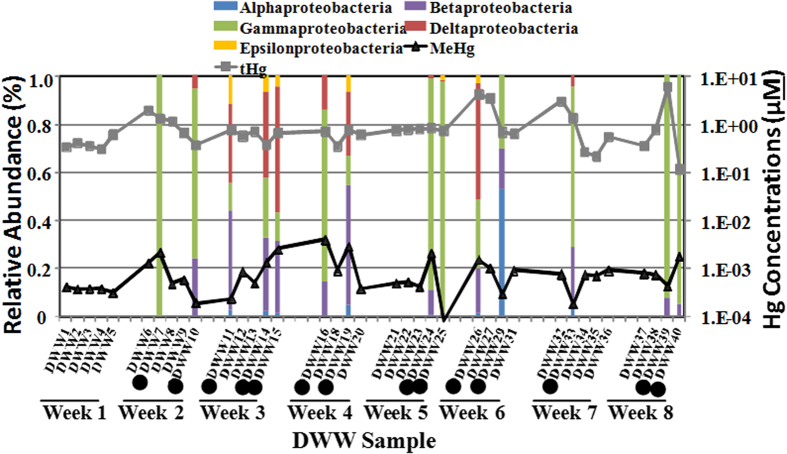
Relationship between proteobacterial diversity and tHg and MeHg over time in DWW samples (DWW1-DWW40) collected from the 12-chair dental clinic. Primary vertical axis reflects the variations in relative abundance of Proteobacteria diversity, and the secondary vertical axis shows the variation in tHg and MeHg concentration during the 8 week sample collection period. (•) Represents samples further selected for pyrosequencing. MeHg at DWW25 (8.1E-5 μM) is not shown for clarity.

F^−^, Br^−^, phosphate and nitrate levels were frequently observed below the detection limit (BDL). Interestingly, sulfate levels increased with decreasing MeHg levels (but the trend was not statistically significant). The sulfate levels were less variable compared to sulfide levels in all the samples and were positively correlated with each other *(r* = 0.55, *P* < 0.05; Table 2). No significant difference was observed among the groups (Supplementary Figure S1a–d). The pH was lowest in DWW14 (6.9) and highest in DWW11 (8.2). All samples with high MeHg had low pH (6.9–7.5) except DWW19 (7.8); whereas all samples with low MeHg levels had high pH levels >7.8 (Supplementary Figure S1e). In addition, significant correlations were observed between the pH and MeHg levels (*r* = -0.64, *P* < 0.01, Table 2, Fig. 2a,b), whereas no correlation was observed with tHg levels. Total Hg levels were correlated with MeHg/tHg ratios and neutral bioavailable Hg(HS)_2_ species (*P* < 0.05, Table 2). As expected, the pH was correlated with neutral bioavailable Hg(HS)_2_ species in DWW (*r* = 0.52, *P* < 0.05, Table 2). Clinic activity during the study period was also variable, ranging from 1–4 fillings and 2–9 surfaces on sampling days during clinic operations (Table 1). In general, Ag and Cu did not vary significantly with day of week or with number of fillings, while tHg varied with clinic usage (Supplementary Figure S2a–h). Both MeHg and tHg exhibited significant temporal differences both during the study, and with the day of the week. MeHg levels were high in weeks 3 and 4, and tHg levels were high in weeks 6, 7 and 8 (Fig. 3). The levels of MeHg were high on Tuesdays and Thursdays of the week and tHg levels were high on Mondays and Tuesdays of the week (Supplementary Figure S2c). Surprisingly, MeHg was not correlated with clinic usage (Supplementary Figure S2g), while tHg was strongly correlated with the clinic usage (Supplementary Figure S2d,h). As expected, tHg was highly correlated (*P* < 0.001) with the number of fillings for all days of the week.

**Figure 4:**
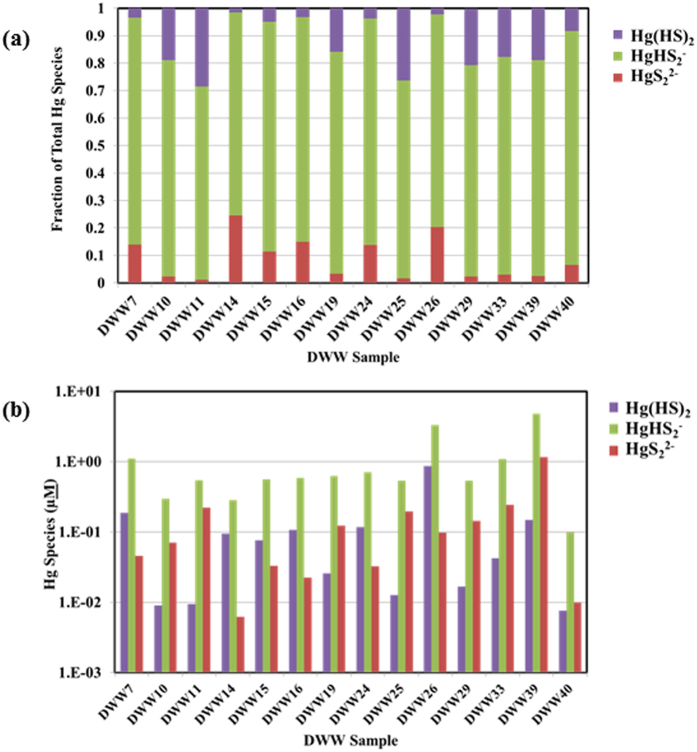
Speciation modelling in DWW samples. (**a**) Fraction of total Hg species, and (**b**) Hg species as predicted by equilibrium Hg geochemical speciation modeling in DWW samples.

Interestingly, equilibrium speciation modeling data suggests that anionic HgHS_2_^−^ and HgS_2_^2−^ complexes (non-bioavailable forms) were dominant in samples with high MeHg levels compared to samples with low MeHg levels (Fig. 4a), and thus Hg would appear to be less bioavailable to bacteria in high MeHg DWW samples. However, when we look at the actual predicted concentration of the neutral bioavailable Hg(HS)_2_ species in these samples (Fig. 4b), a clear demarcation is observed where all high MeHg samples have Hg(HS)_2_ concentrations near or above 0.1 μM, while all low MeHg samples have concentrations at or below 0.01 μM. This strongly suggests that it is the actual concentration of the neutral species that is important, not just the fraction of the total. Although studies have shown positive correlations between DOC and Hg methylation under sulfidic conditions (possibly suggesting DOC-mediated abiotic methylation)^22^, we did not observe such a correlation in DWW.

### General sequencing statistics and DWW bacterial community composition

We performed Tag-encoded FLX amplicon pyrosequencing screens on the 14 DWW subset samples to characterize the bacterial community composition and phylogenetic diversity in DWW. For the 454 pyrosequencing, 14 DWW samples yielded a total of 110,292 sequencing reads. Following the quality trimming protocol, 85,000 high quality reads (>300 bp) were RDP-classified as bacteria, four reads classified as Archaea and 11 reads as unclassified roots. A total of 13,040 reads could not be identified and remained as unclassified bacteria. An additional 23,000 sequences (21%, <300 bp) were short in length and were removed before further analysis. Employing deep sequencing of 16S rRNA gene amplicons, we estimated the bacterial diversity of the DWW microbiome.

The total DWW bacterial community contained 24 phyla, unclassified bacteria, and Archaea from the sequencing data. Sequences which showed less than 80% homology to the unclassified bacteria constituted 26% of the microbial community. The obtained taxonomy data demonstrates that DWW is remarkably biodiverse; covering a broad spectrum of known and unknown bacterial taxa (Table 3). The bacterial community consisted of Proteobacteria (21 to 98%), unclassified Bacteria (0.4 to 96%), Firmicutes (0.1 to 74%), Cyanobacteria (0.02 to 15%), Bacteroidetes (0.2 to 12%), Chloroflexi (0.3 to 7%), Fusobacteria (0.2 to 6%), Actinobacteria (0.2 to 3%), Synergistetes (0.04 to 2%) and Acidobacteria (0.02 to 1%). The remaining 16 phyla were grouped as other phyla, which were detected in lower abundance (<1%) in 10 out of 14 DWW samples. A total of 170 bacterial taxa, 58 unclassified families and unclassified bacteria were identified in 14 DWW samples. The dominant phyla in all samples belonged to Proteobacteria and were detected in all 14 DWW samples studied. They were apportioned as Gammaproteobacteria (1.5 to 98%, detected in all 14 samples), Deltaproteobacteria (0.1 to 39%, detected in 12 out of 14 samples), Betaproteobacteria (0.6 to 38%, detected in 13 out of 14 samples), Alphaproteobacteria (0.1 to 27% detected in 13 out of 14 samples) and Epsilonproteobacteria (1.8 to 7.7%) was the least common of the Proteobacteria, found in only six out of 14 samples. A total of 2,300 reads could not be classified below Proteobacterial class level and remained as unclassified Proteobacteria accounting for ~4% of the total Proteobacteria.

Comparing the eight most abundant bacterial families (Table 3) and dominant predicted methylators in each sample (Table 4,^33^) reveals large taxonomic differences between the DWW samples. The families Rhodocyclaceae (10–35%), Xanthomonadaceae (30–90%) and the SRB (Desulfovibrionaceae and Desulfobulbaceae) (5–25%) were highly abundant in high MeHg samples, and either absent or less common in low MeHg DWW samples (Table 3). Given the very high levels of potentially bacterio-toxic metals like tHg (>6 μM), Cu (>350 μM), Zn (>150 μM) and Ag (>20 μM) in DWW (Supplementary Table S1), these results suggest that metal-resistance is phylogenetically-broadly distributed among DWW bacterial communities.

### Proteobacteria diversity

Alphaproteobacterial diversity was lowest in all DWW compared to other classes of Proteobacteria. DWW29 had the highest (27%) Proteobacteria diversity compared to other DWW samples. Alphaproteobacteria were detected in all DWW samples except DWW39. A total of 11 families with unclassified Alphaproteobacteria and 28 genera were detected in all DWW samples. Betaproteobacteria were dominant in DWW11 and DWW19 (28 to 38%), and were detected in all 14 DWW samples except DWW7. A total of eight Betaproteobacterial families were identified with 31 genera and unclassified Betaproteobacteria. In all DWW samples, Betaproteobacteria and Alphaproteobacteria were found to be more evenly distributed among samples. Gammaproteobacteria were detected in all DWW samples as the dominant class of bacteria (1.5 to 98%) in the total bacterial community. They were abundant in DWW7 (98% of total Bacteria), DWW25 (95% of total Bacteria) and DWW40 (92% of total Bacteria). Deltaproteobacteria were the second most dominant class of Proteobacteria in the DWW. These results are consistent with the hypothesis that SRB are important methylators in DWW, as virtually all known Hg methylators detected are in the Deltaproteobacteria (Table 4). DWW15 had the largest proportion of Deltaproteobacteria (39% of the total Bacteria), followed by DWW26 (35% of total Bacteria). DWW14 and DWW11 had approximately equal proportions (~22%) of total Bacteria. The samples with high MeHg levels had higher abundances of Deltaproteobacteria (12 to 42%) and Epsilonproteobacteria (5 to 22%), compared to the samples with low MeHg levels (except DWW11 which had higher abundance of both groups). No Deltaproteobacterial reads were detected in DWW7 and DWW39 (the former was classified as a high MeHg sample). A total of 15 Deltaproteobacteria families were identified representing 26 genera in the DWW samples: Desulfovibrionaceae, Desulfobulbaceae, Syntrophobacteraceae and Desulfobacteraceae. The dominant genera identified are *Desulfovibrio*, *Desulfobulbus*, *Desulforhabdus*, unclassified Desulfovibrionaceae, unclassified Desulfobacteraceae, *Desulfomonile*, *Geobacter*, *Pelobacter* and *Syntrophus*. In DWW16, (the sample with the highest MeHg levels) *Desulfovibrio* sp. were identified as the dominant SRB species, which include many known Hg methylators (Table 4). Overall, the abundances of Epsilonproteobacteria were low (2 to 8%), and these bacteria were detected in 6 out of 14 DWW samples (DWW11, DWW19, DWW14, DWW15, DWW26 and DWW25, in order of high to low Epsilonproteobacteria sequence reads). Genera detected in these samples include *Sulfurospirillum*, *Campylobacter*, *Sulfuricurvum*, and *Sulfurimonas*. Details of the other important bacterial groups identified in 14 DWW samples are given in the supplementary data. Correlations between MeHg, tHg geochemical characteristics and microbial diversity at different taxonomic level were also studied. Significant Pearson correlations among environmental variables and microbial taxa at different levels are shown in Table 5.

### Diversity patterns in the DWW bacterial communities are linked with water geochemistry

**Figure 5:**
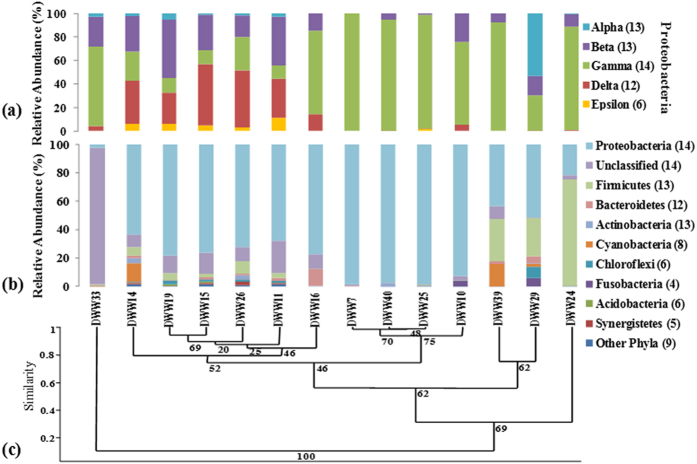
Comparison of bacterial diversity between the different DWW samples. (**a**) Stacked column graph representing relative percent abundance of classes within the phylum Proteobacteria. (**b**) Stacked column graph representing the relative percent distribution of the dominant phyla in the 14 DWW samples. Values in parentheses corresponds to the number of samples (out of 14) in which that particular phyla or class were detected. (**c**) Cluster analysis of bacterial diversity at the phylum level using the Bray-Curtis matrix of similarity. Low abundance phyla are grouped together as “other phyla” and their cumulative abundances are listed.

The microbial community composition in the 14 DWW sample subset was evaluated using Cluster analysis (CA) and Principal Coordinate Analysis (PCoA). Hierarchical cluster analysis based on the Bray-Curtis (BC) similarity matrices for the bacterial community composition at the phylum and class level revealed sample specific differences (Fig. 5a–c). Cluster analysis at the class (Fig. 5a), and phylum level (Fig. 5b) showed conservation of community composition in DWW14, DWW19, DWW15, DWW26, DWW11 and DWW16. These samples were similar to each other and clustered separately from DWW33 and three other clusters. These samples had the highest proportions of Betaproteobacteria and Deltaproteobacteria groups. In contrast, DWW33 had the highest percentage of 16S rRNA gene sequences not clustered into any known taxonomic group, and was dominated by unclassified bacteria (>95% of total bacteria). DWW7, DWW40, DWW25, and DWW10 were similar in community composition and clustered separately from DWW39, DWW24 and DWW29. DWW7, DWW40 and DWW25 had the largest proportions of Gammaproteobacteria (92 to 98%), and the lowest proportions of unclassified bacteria (0.01 to 0.4%). DWW39 (a Wednesday sample) had the largest proportion of Cyanobacteria, which were not detected in DWW7, DWW40, DWW25, and DWW10. DWW39, DWW29 and DWW24 had high proportions of Firmicutes and other bacterial groups. It is not known how cyanobacteria (a photosynthetic microorganism) were present in the dark environment of the DWW sewer. This was a mid-week sample and may have included DWW that was recently produced from the clinic. Interestingly, Alphaproteobacteria, Betaproteobacteria, Deltaproteobacteria and Epsilonproteobacteria clustered together in high MeHg samples, whereas Gammaproteobacteria were clustered together in low MeHg samples. Together, these results demonstrate that there is distinct clustering of the microbial communities between those samples with high MeHg and those with low MeHg.

**Figure 6:**
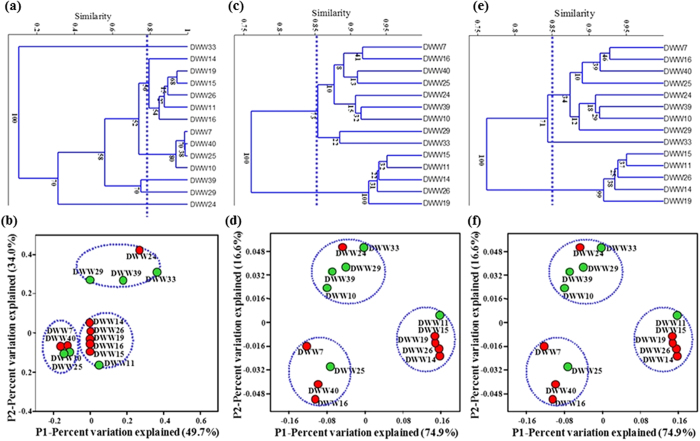
Cluster and principle coordinate analysis of 14 DWW samples. Cluster analysis are shown at phylum level (**a**), at the class level (**c**), and at the genus level (**e**). The analysis is conducted using the unweighted pair group mean averages (UPGMA) based on the Bray-Curtis distance. Dotted lines show the similarity cutoff level to cluster and group 14 DWW samples. The principle coordinate analysis are shown at phylum level (**b**), at the class level (**d**), and at the genus level (**f**). Filled red circles represent samples with high MeHg and green circles represent samples with high tHg and low MeHg levels. Note different x axis scales.

CA and PCoA analysis further revealed that the bacterial composition was similar at the phylum level (Fig. 6a,b) but slightly changed at the class (Fig. 6c,d), and genus level (Fig. 6e,f). The PCoA analysis (Fig. 6b,d,f) results showed consistent clustering at the class and genus level, as was observed in the cluster analysis (Fig. 6a,c,e). DWW11 and DWW25 did not follow this trend and grouped together in PCoA analysis with high MeHg samples (Fig. 6d–f). Similarly, DWW24 grouped with low MeHg samples even though it had higher MeHg. These clustering patterns demonstrate agreement between both the CA and PCoA distance metric results.

### Canonical correspondence analysis (CCA)

**Figure 7:**
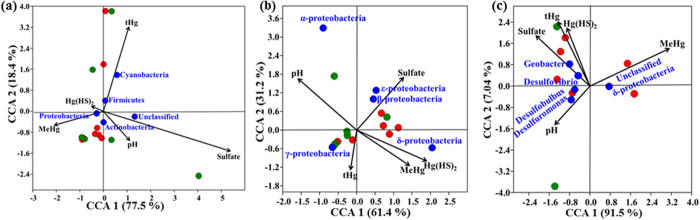
Canonical correspondence analysis plots. First two axes of CCA1 and CCA2 represent the relationships between environmental variables and bacterial diversity at (**a**) phylum level, (**b**) class level for Proteobacteria taxonomic class, (**c**) genus level (mercury methylating Deltaproteobacteria groups). Symbols indicate phyla/classes/genus (blue dots), high MeHg samples (red dots), low MeHg samples (green dots) and environmental variables (black arrows). Arrows indicate the direction and magnitude of measurable variables associated with bacterial community structures. The percentage of variation explained by each axis is shown in axis label.

CCA was performed to identify major environmental parameters linking to DWW microbial community at different taxonomic levels. According to the CCA, the effects of individual environmental factors on DWW microbiome varied across the samples. MeHg, tHg, Hg(HS)_2_, pH and sulfate levels were the most important parameters shaping the overall microbial community. The first two axes explained 95% of the taxonomic information at phylum level (Fig. 7a). The length of the arrow of environmental parameter in the ordination plot indicates the strength of the relationship of the parameter to the DWW community composition. CCA in phylum-level diversity indicated that the phylum Cyanobacteria was tightly associated with the tHg, the phylum Actinobacteria was associated with the Hg(HS)_2_ and pH, and Proteobacteria was correlated with MeHg levels (Fig. 7a). Classes of Beta and Epsilonproteobacteria were associated with the sulfate levels, Alphaproteobacteria with pH*-* and Gammaproteobacteria were correlated with tHg levels. Deltaproteobacteria were associated with both the MeHg and Hg(HS)_2_ levels in DWW. The first two axes explained 92% of the taxonomic information at Proteobacteria class level (Fig. 7b). CCA in genus level diversity of mercury methylating Deltaproteobacteria group indicated that *Desulfobulbus* and *Desulfuromonas* were correlated with pH, *Geobacter* and *Desulfovibrio* were associated with tHg, Hg(HS)_2_ and sulfate levels and unclassified Deltaproteobacteria with MeHg levels (Fig. 7c).

**Figure 8:**
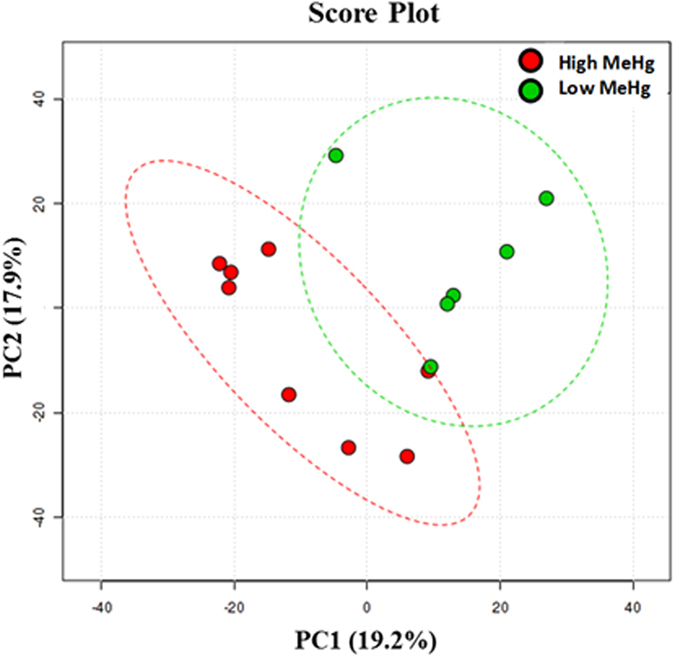
Microbial diversity at genus level reveals hierarchical partitioning of high and low MeHg DWW samples. Bacterial communities were clustered using partial least squares-discriminant analysis (PLS-DA). Red circles represent samples with high methyl Hg levels and green circles represent samples with low methyl Hg.

Mantel test analysis also revealed significant correlations between microbial community structure and different environmental factors. Different parameters such as number of OTUs, (*r* = 0.38, *P* = 0.06) were correlated with Bray-Curtis dissimilarity matrix for Proteobacteria class. Hg methylating Deltaproteobacteria members *Desulfobulbus* (MeHg/pH/tHg, *r* = 0.36, *P* = 0.05), unclassified Deltaproteobacteria (MeHg, *r* = 0.53, *P* = 0.1) and *Geobacter* species (pH, *r* = 0.31, *P* = 0.04; tHg, *r* = 0.34, *P* = 0.06) were highly correlated with pH, MeHg, and tHg. Overall, the consensus for different parameters revealed that pH, MeHg, tHg and sulfate were the major factor affecting microbial taxon abundance in different DWW samples (Supplementary Table S2). Partial least squares-discriminant analysis (PLS-DA) also illustrated a distinct microbial diversity segregation at the genus level for the DWW microbiome primarily related to geochemical factors and MeHg conditions (Fig. 8).

### Similarity Percentage (SIMPER) analysis

Since a difference in the bacterial community was observed between the high and low MeHg group, SIMPER analysis was performed to show which taxa contributed to this difference the most. The SIMPER results for main taxa are shown in Table 6. The taxa that contributed the most of the differences at phylum level were Proteobacteria (16.7% avg. dissimilarity), unclassified bacteria (10.8% avg. dissimilarity) Firmicutes (8.6% avg. dissimilarity) and Cyanobacteria (2% avg. dissimilarity). In particular, Proteobacteria and Firmicutes were highly abundant in the high MeHg samples, whereas unclassified bacteria and Cyanobacteria were abundant in low MeHg samples. Together, these taxa accounted for 41.6% of the difference. Rest of the four phyla accounted for 0.5 to 1% of the difference (Table 6). The high contribution of Proteobacteria is of particular interest, because it was the dominant taxa in all the DWW samples, SIMPER analysis was also performed at Proteobacteria class level.

**Figure 9:**
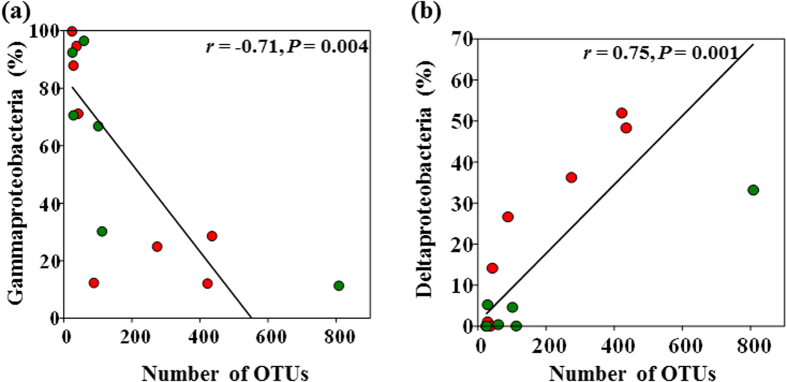
Linear regressions were used to test Pearson’s correlation between number of OTUs detected for Gamma- and Deltaproteobacteria in high and low MeHg DWW samples. (**a**) The correlations between number of OTUs and relative abundance of Gammaproteobacteria. (**b**) OTUs and relative abundance of Deltaproteobacteria in DWW. Pearson correlation coefficient and *P* values are shown. Red and green dots represent high and low MeHg samples, respectively.

Gammaproteobacteria (19.2% avg. dissimilarity) and Deltaproteobacteria (10.7% avg. dissimilarity) contributed the most of the difference, followed by Beta- (8.1%) Alpha- (4.9%) and Epsilonproteobacteria (1.8%). Overall, 42.7% difference was accounted by these classes. Gamma and Betaproteobacteria were abundant in both the groups, whereas Deltaproteobacteria were dominant in high and Alphaproteobacteria were dominant in low MeHg group. These results revealed that the abundance of Proteobacteria class had a high influence on dissimilarities observed among the groups. Linear regressions also showed a high correlation with number of OTUs detected for Gammaproteobacteria (*r* = 0.71, *P* < 0.001, Fig. 9a), and Deltaproteobacteria (*r* = 0.75, *P* < 0.001, Fig. 9b), in high and low MeHg samples.

### Fold change analysis

**Figure 10:**
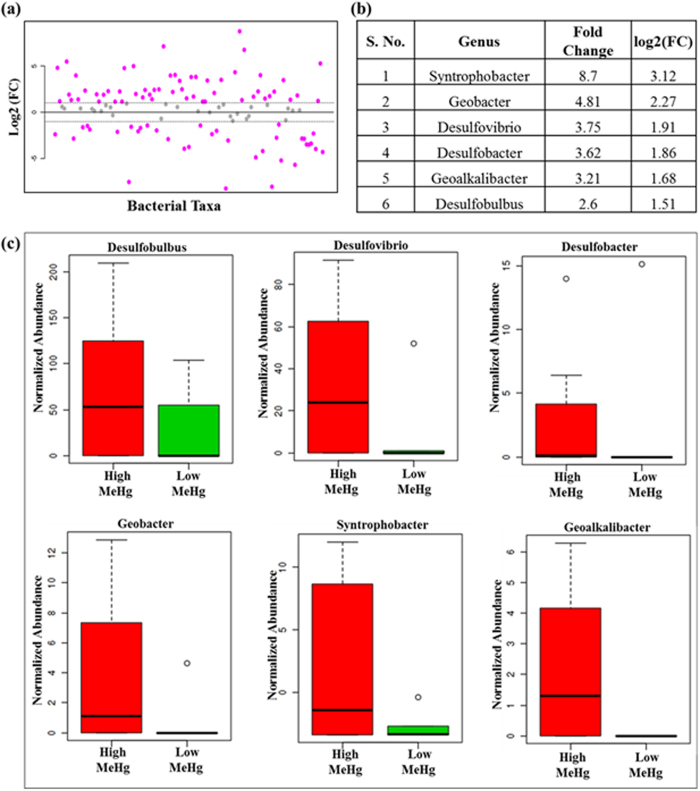
Fold change (FC) analysis between high and low MeHg group means. (**a**) FC analysis of the bacterial taxa abundance between the high and low MeHg samples (the effect size and direction of the correlation is presented by the FC value and color). (**b**) The FC value of the significant correlations for important mercury methylating groups. (**c**) The FC values were calculated as ratio between high and low MeHg group means using normalized data. Shown are box and whisker plots of bacterial taxa abundance change in important Hg methylating bacterial taxa.

Nearly half (46%) of the total bacterial taxa exhibits a fold change (FC) greater than 1.5 (Fig. 10a). These results clearly illustrate that the high levels of MeHg, tHg and other heavy metals are primarily responsible for changes in relative community abundance. More specifically, in the high MeHg group, important mercury methylating bacteria (*Desulfobulbus, Desulfovibrio, Desulfobacter, Geobacter, Syntrophobacter* and *Geoalkalibacter*) significantly increased by 2.6–8.7% units (1.8–3.1 fold change) in Fig. 10b. Figure 10c, represents the box and whisker plots of bacterial taxa abundance change in known Hg methylating bacteria. Clear and highly statistically significant differences are observed between the levels of each class of known methylators in high and low MeHg samples. The fold change values were calculated as the ratio between high and low MeHg group means using normalized data (Supplementary Figure S2).

### Mercury resistant bacterial taxa

In DWW we detected the presence of nine mercury resistant bacterial genus that have been reported to tolerate and resist high mercury levels, representing the following genera - *Stenotrophomonas, Staphylococcus, Pseudomonas, Tolumonas, Escherichia, Aeromonas, Bacillus, Burkholderia*, and *Streptococcus*. In all DWW samples *Stenotrophomonas* were abundant (25% of total). *Staphylococcus* were more represented in high MeHg group compared to low. *Pseudomonas* and *Escherichia* were slightly higher in low MeHg samples. The percent abundance of these bacterial species was variable among the groups (Table 7a). We further performed the Pearson correlation and found *Stenotrophomonas, Staphylococcus, Pseudomonas, Tolumonas, Escherichia, Aeromonas, Bacillus, Burkholderia* were correlated with different geo-chemical parameters such as, pH, tHg, MeHg/tHg, sulfate and sulfide levels (Table 7b).

There was no statistically significant correlation observed (student’s *t*-test, *P* = 0.8) among the mercury resistant bacteria (genus level) in high and low MeHg samples. Also no significant correlation (*r* = −0.3, *P* = 0.4) was observed among the tHg and total abundance of mercury resistant bacteria in DWW (Supplementary Table S2). However, Mantel test showed a weak association in Bray-Curtis matrix of dissimilarity in mercury resistant taxa and Euclidean distance matrix of environmental variables. *Stenotrophomonas* (pH/tHg/MeHg, *r* = 0.42, *P* = 0.04), and *Pseudomonas* (MeHg, *r* = 0.15, *P* = 0.02) were significantly correlated with some of the geochemical parameters (Supplementary Table S2).

### DWW microbial Community richness and diversity

We grouped 16S rRNA gene sequences with their nearest neighbours as shown by BLASTn and RDP searches. Clone clusters are comprised of one or more operational taxonomic units (OTUs)/phylotypes, and sequences with more than 97% similarity were considered to be the same OTU and were clustered together to calculate rarefaction and nonparametric estimators. The frequencies of the OTUs obtained are shown (Table 8). Diversity index quantifies the diversity in a community and describes its numerical structure. The analysis demonstrates that bacterial diversity in the 14 DWW samples have been sufficiently covered by pyrosequencing (Table 8). A total of 2,546 OTUs were obtained from 14 DWW samples. Clustering the unique sequences into OTUs resulted in 24 to 808 different species-level OTUs per DWW sample. Diversity indices calculated for each of 14 DWW samples ranged from 1.0 to 4.8 (Shannon index) and from 34 to 631.5 (Chao1 index). The highest bacterial richness and diversity were found in DWW11 and DWW26 and were lowest in DWW24 and DWW7. The Shannon diversity index values and Chao1 richness estimates for the DWW11 were 4.8 and 631, and 1.0 and 37 for DWW7 respectively. Together, the number of OTUs, Chao 1, ACE, and Shannon indices indicate that the species richness varied by 2–3 times among the different DWW samples (Supplementary Figure S4a–c). The OTU abundances and Shannon index based on both ARISA and pyrosequencing had a similar trend in the 14 DWW samples (Supplementary Figure S5a).

Species evenness (*E*) for the DWW samples ranged from 0.3 to 0.7 (Table 8). Species evenness was highest in DWW19 and DWW26 (0.74 and 0.73 respectively) and lowest in DWW24 and DWW7 (0.31 and 0.32 respectively). The sample coverage using Good’s method was 88 to 99%. In high MeHg samples, OTUs and Shannon indices were high (24 to 435 and 1 to 4.5, respectively), whereas in low MeHg samples they were low (27–112 and 1 to 3 respectively). The lone exception to this trend was DWW11 (808 and 4.8, respectively). These values indicate that the diversity and evenness are high in DWW samples, in contrast to our expectations that the DWW would be dominated by SRB methylators and a few metal resistant bacterial species. Rarefaction analysis of the bacterial diversity in all DWW samples revealed four curves that did level off but not all had fully reached an asymptote (Supplementary Figure S5b). Three of these four were high MeHg containing samples (DWW 14, 15 and 26). This indicates that additional species diversity may be present in these samples, consistent with the quantitative diversity estimator analysis indicating that high MeHg samples were more biodiverse.

### Phylogenetic analysis

**Figure 11:**
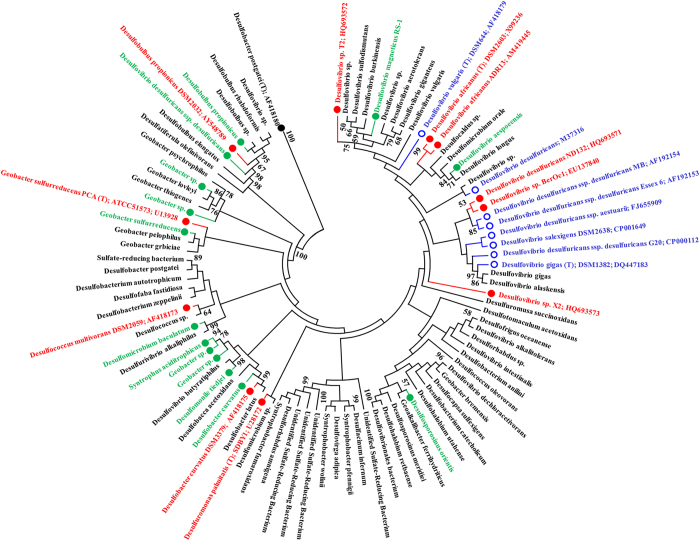
16S rRNA gene phylogeny for the predicted methylators identified in DWW samples. Type strains are indicated by – ‘T’. *Desulfobacter postgatei* was used as a reference and outgroup taxon. Strains reported to produce MeHg are shown with a filled red circle 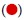, those unable to do so are shown with an open blue circle 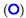, and strains with a filled green circle 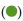 are predicted methylators (from this study). The evolutionary history was inferred using the neighbor-joining method and the evolutionary analyses were conducted in MEGA.

Bacterial species known to produce MeHg to date are in the class Deltaproteobacteria, but only about half of the species tested so far have the ability to produce MeHg. We constructed a 16S rRNA gene-based phylogeny of the class Deltaproteobacteria to explore the potential Hg methylating bacteria within DWW (Fig. 11). Our alignment included the 79 representative OTUs identified within class Deltaproteobacteria from DWW and 19 reference strains for which Hg methylation has been tested and for which sequences were available in the literature (strain *D. desulfuricans* LS could not be included in analysis). Among the Deltaproteobacteria, *Desulfovibrio* is a large and diverse genus; and thus all of the *Desulfovibrio* type strains could not be included in the tree.

The analysis yielded well-supported deep branching main clades and a number of less well-supported smaller groups. Most of the well-studied Hg methylating *Desulfovibrio desulfuricans* strains, including *D. desulfuricans* strains Essex 6, MB, *estuarii* and G20, fall into this group. Most of the strains tested for methylation to date fall in this group. This clade includes another closely related group of strong Hg methylators, strain ND132 and *Desulfovibrio* strain BerOc1. However, the group is also closely related to *D. desulfuricans* strain E1 Agheila (Accession number: M37316), which does not have the ability to produce MeHg. Weakly related to this group is *D. africanus*; both the type strain and strain ADR13, are Hg methylators. They are both weakly related to *D. vulgaris* type strain, (a non Hg methylating strain) and Hg methylating strain T2 from Chesapeake Bay. The strain T2 clusters closely together with eight representative *Desulfovibrio* species identified within DWW. This group of species with the ability to utilize a broader set of substrates clusters loosely together. This group contains Hg methylating strain X2 which clusters closely together with many Deltaproteobacteria species identified in DWW. To date, no other species in this group have been tested for Hg methylation potential. *Desulfuromonas palmitatis* type strain SDBY1 and *Desulfobacter curvatus* strain DSM3379 are both known Hg methylating species and they clustered closely together with other *Desulfuromonas* and *Desulfobacter* species identified in DWW. *Desulfococcus multivorans* DSM2059 (a known Hg methylating strain) clustered closely together with other *Desulfococcus* and related species identified in DWW. Interestingly, this group is not closely related to another Hg methylating strain *Geobacter sulfurreducens* PCA, which clusters closely with many *Geobacter* spp. identified in DWW. *Desulfobulbus propionicus* DSM2032 Hg methylating strain is clustered closely with other *Desulfobulbus* species identified in DWW.

Recent studies have shown that most of the commonly studied *Desulfovibrio* strains align into a clade with few Hg methylators, while most of the Hg methylating strains fall into the halophiles group^20^. The finished genomes for *Desulfovibrio* strain ND132, *Desulfovibrio africanus* strain Walvis Bay and *Desulfovibrio africanus* strain PCS are the first complete genomes for the strains that generate MeHg^38^,^39^,^40^. This will aid in to our studies with comparison of full genomes of known Hg methylators identified in DWW and comparative metagenomics of Hg contaminated ecosystems.

## Discussion

The goal of present study was to determine how microbial community composition varies across high and low MeHg DWW samples from a dental clinic, and to determine any associations between these communities and Hg methylation potential. Our study investigates the potential of Hg in dental-amalgam and other possibly bacterio-toxic heavy metals like Ag, Zn and Cu to impact the composition of microorganisms within the DWW holding tank, where Hg levels are highest. Our analysis shows associations between tHg, MeHg, geochemical parameters and the composition of the microbial community. The results indicated that the microbial communities are complex and highly dynamic, correlate strongly with MeHg levels, and geochemical data are consistent with SRB-mediated methylation in DWW. Equilibrium speciation data suggested a clear demarcation where all high MeHg samples have high concentrations of the neutral Hg species compared to all low MeHg samples, suggesting that concentration of bioavailable Hg species is important, not just the fraction. Pearson correlation matrix analysis for geochemical characteristics showed that pH was significantly correlated with MeHg, and tHg and MeHg were correlated with neutral bioavailable Hg species. The pH is typically the most important environmental factor determining the speciation of metals. The pH has been found to dramatically affect the amount of sulfide-bound Hg available for uptake in the non-ionic Hg(HS)_2_ as well as changes the binding energy of Hg to organic matter^41^,^42^. The low pH observed in high MeHg DWW samples suggests that Hg is both bioavailable and accessible to resident microbial communities for methylation. CCA and Mantel test analysis revealed significant correlations between microbial community structure and water geochemistry. Hg methylating bacteria were correlated with pH and Hg levels. Overall, the consensus for different parameters revealed that pH, MeHg, tHg and sulfate were the major factors affecting microbial taxon abundance in DWW. SIMPER analysis identified that Proteobacteria, unclassified bacteria, Firmicutes and Cyanobacteria contributed most of the dissimilarity among the high and low MeHg group. Among Proteobacteria, Gammaproteobacteria and Deltaproteobacteria contributed the most to these differences. These results also suggest that DWW bacteria can tolerate high levels of tHg and MeHg, as well as high levels of Ag, Cu and Zn; and thus wide spectrum heavy metal resistance may play a role in Hg methylation in DWW. High MeHg samples were distinct both geochemically and in microbial community composition, particularly in the abundance of SRB. In the high MeHg group, important mercury methylating bacteria *Desulfobulbus, Desulfovibrio, Desulfobacter, Geobacter, Syntrophobacter* and *Geoalkalibacter* were significantly high compared to low MeHg samples.

Given the high levels of tHg, MeHg, Ag, Cu, and Zn, it is not surprising that metal resistance may have had a significant and complex influence on the microbial community. Epsilonproteobacteria have been shown previously to associate with Hg, in our study they show an association with sulfate levels. Some members of this class include sulfur-oxidizing bacteria like *Sulfurimonas*, *Sulfurospirillum*, *Campylobacter* and *Sulfuricurvum*. These bacteria are found in extreme environments and genome sequences of *Sulfurimonas* strain have revealed genes similar to those found in mercury resistance (*mer*)^43^. Our sequencing data revealed approximately 1,500 sequences related to this group. Previous studies have linked the methylation process to increasing SRB activity. Whether this also explains the abundance of other bacterial groups in DWW samples is unknown at this time.

In the DWW microbiome, we detected nine mercury resistant bacterial genera that have been reported in the literature to tolerate high mercury levels^34^,^44^,^45^. There was no statistically significant difference between the relative abundance of mercury resistant bacteria in high and low MeHg samples, and no correlation with levels of Hg was detected. Overall, a weak association was observed between geochemical parameters and mercury resistant taxa. However, *Stenotrophomonas* and *Pseudomonas* were significantly correlated with some geochemical parameters.

Hg resistant microbes have been isolated from the high Arctic, belonging to the Alpha-, Beta- and Gammaproteobacteria, Firmicutes, Actinobacteria, and Bacteriodetes^46^. The resistance to inorganic and organic mercury compounds is mediated by the mercury resistance loci in Gram-positive or Gram-negative bacteria. This loci is comprised of *merA*, or/and *merB, merT and merR*^36^,^45^. Hg resistance by the reduction of mercuric to ionic Hg is broadly distributed among bacteria, and plays an important role in Hg detoxification and biogeochemical cycling^36^,^47^,^48^. Bacterial resistance to MeHg is encoded by the two enzymes, mercuric reductase (*MerA*) and organomercurial lyase (*MerB*)^36^,^37^,^45^,^49^. Mercuric resistance gene clusters are associated with other metal resistance genes^50^. The heavy metal resistance and *mer* genes have been studied in many bacteria including *Bacillus, Clostridium, E. coli, Enterococcus, Staphylococcus, Streptococcus, Streptomyces, Shewanella, Klebsiella, Pseudomonas, Pseudoalteromonas*, and many other Gram-negative and Gram-positive bacteria^37^,^51^,^52^,^53^. Several known metal and sulfate reducing bacteria (*Geobacter*, *Desulfovibrio* and *Desulfitobacterium*) have been reported in heavy metal contaminated water samples^54^. The high microbial community diversity in the DWW microbiome is consistent with the idea that heavy metal resistance are more widely distributed than previously thought, possibly through natural resistance by glutathione that is known to offer protection from various metals including Ag, Cu, and Hg^55^,^56^. In our results, SRB and FeRB were abundant in DWW, indicating that they have successfully adapted to the DWW environment. This supports the hypothesis that both SRB and FeRB contribute to MeHg production in the DWW. This does not rule out the possibility that other types of bacteria may also be responsible for Hg methylation in DWW.

Recent studies have used comparative genomics and targeted gene deletion to identify two candidate genes *hgcA* (which encodes a putative corrinoid protein) and *hgcB* (which encodes a 2[4Fe-4S] ferredoxin), which are required for mercury methylation, but are absent in non-methylators. This suggests a common mercury methylation pathway in all methylating bacteria^33^,^57^. In our study, we have identified Hg methylating bacteria in DWW microbiome known to harbor the required gene cluster of *hgcAB* for mercury methylation. Our results also revealed that strong Hg methylators such as *Desulfobulbus propionicus*, *Geobacter sulfurreducens*, and *Desulfobacter* sp. were highly abundant in high MeHg group.

Further, our results indicate that Hg methylation occurs over sustained time periods in DWW and is not episodic. However, the specific mechanism for high MeHg levels (increased methylation, decreased demethylation, or a combination of the two) may have varied within each sample and thus is not ascertained at this time. In addition there is a gap between phylogeny and the distribution of *hgcA* and *hgcB* among various species, which limits our ability to predict methylation activity based on phylogenetic relationships. Metagenomic analysis and genetic probes targeting specific genes should significantly increase the reliability for predicting net methylation. Thus, our study shows a more comprehensive understanding of DWW microbial community dynamics through metagenomic analysis. More broadly, the diversity of the microbial community structure in these highly-polluted environments suggests the need for broadening our view of Hg methylation and metal resistance, as these capabilities may be more phylogenetically widespread than previously thought. Alternatively, the high biodiversity of these environments may be the key to why so many bacterial species can withstand such high levels of heavy metals.

## Methods

### Ethics statement

All study procedures, experiments and methods were carried out in accordance with the approved guidelines by the Institutional Review Board, University of Illinois, Chicago, IL, USA, under Protocol #2006-0137. All the experimental methods have been approved by Institutional Review Board, University of Illinois at Chicago and informed consent was obtained from all subjects.

### DWW sample collection and analysis

A total of 40 DWW samples were collected from the 12-chair dental clinic at the College of Dentistry, University of Illinois, Chicago, IL, over an eight week period. All amalgam fillings placed, replaced or removed were recorded during the entire sampling period. The sample collection, characterization and equilibrium speciation modeling details are described in the supplementary data.

### Heavy metals and mercury analysis

Samples for metal analysis were prepared according to USEPA method SW 3050B and all metals except Hg were analyzed by inductively coupled plasma-mass spectrometry using USEPA method SW 6020A^58^. Total Hg and MeHg were analyzed with the method described previously^13^,^59^. Total Hg was quantified by USEPA standard method 1631 and 1630^60^,^61^ using a Brooks-Rand Cold Vapor Atomic Fluorescence Spectrometry (CVAFS) System (model III, Brooks-Rand, Seattle, WA). Method and quality assurance details are described in the supplementary data.

### DNA extraction and automated ribosomal intergenic spacer analysis (ARISA) of DWW microbiome

DNA isolation and quantification was performed as described previously^13^. ARISA was used initially for the estimation of microbial diversity and community composition in DWW samples. This approach aided in selecting important samples for pyrosequencing and allowed a rapid comparison of the large number of samples. The method is described in the supplementary data.

### 16S rRNA gene tag sequencing and data analysis

Bacterial tag-encoded FLX 454 amplicon pyrosequencing (bTEFAP) and data processing were conducted at DNA Services Facility, Research Resource Center, University of Illinois, Chicago, and Research and Testing Laboratory (Lubbock, TX, USA) as described previously^62^,^63^. For this study, PCR amplification was performed using the primers Gray28F and Gray519R (Supplementary Table S3) to span the variable regions (V1–V3) of 16S rRNA gene^64^. Method details are described in the supplementary data.

### Estimates of microbial diversity

Taxonomic classification of the bacterial sequences of 14 DWW samples was carried out individually using the Ribosomal Database Project (RDP) classifier. Bacterial 16S rRNA gene sequences were aligned phylogenetically using the naïve Bayesian rRNA classifier to assign the sequences to different taxonomic levels (http://rdp.cme.msu.edu/classifier/classifier.jsp). Method details are described in the supplementary data.

### Cluster Analysis (CA) and Principal Coordinate Analysis (PCoA)

CA and PCoA were conducted to group the bacterial communities of the different DWW samples on the basis of operational taxonomic units (OTUs) generated using RDP Complete Linkage Clustering. CA and PCoA were conducted using the Bray-Curtis distance calculated from the similarity matrix using the statistical software package PAST v1.82b^65^. CCA and Mantel test was performed using XLSTAT-ADA (Addinsoft, USA) to find the association between geochemical variables and microbial diversity using 1000 permutations and a 5% significance level. Similarity percentage (SIMPER) analysis was used to identify the taxa that were mainly responsible for the differences observed between the high and low MeHg samples. All the analyses were performed with normalized taxa abundance, using the PAST. Analysis details are described in supplementary data.

### Molecular phylogenetic analysis

A 16S rRNA gene-based phylogenetic tree of known Hg methylating bacteria within the class Deltaproteobacteria was constructed. The tree was based on the aligned representative sequences for 78 OTUs (within class Deltaproteobacteria) identified from 14 DWW samples in this study. Nucleotide sequences were aligned using ClustalW and a phylogenetic tree was constructed using MEGA version-4^66^. Method details are described in supplementary data.

### Quality assurance for Hg and metal analysis

Quality assurance was carried out as described previously^12^,^13^ and is discussed in detail in the supplementary information.

## Additional Information

**How to cite this article**: Rani, A. *et al.* Geochemical influences and mercury methylation of a dental wastewater microbiome. *Sci. Rep.* **5**, 12872; doi: 10.1038/srep12872 (2015).

**Accession numbers**: The sequences data were deposited, and are publicly available at the MG-RAST web-server^67^ with the following accession numbers: 4497483.3, 4497956.3, 4497957.3, 4497958.3, 4497959.3, 4497961.3, 4497962.3, 4497963.3, 4497964.3, 4497966.3, 4497968.3, 4497969.3, 4497970.3 and 4497971.3.

## Supplementary Material

Supplementary Information

## Figures and Tables

**Table 1:** Geochemical characteristics of the 14 DWW sample subset selected for pyrosequencing and ARISA analysis.

Sample	pH	Cl^−^(mM)	SO_4_^2−^(mM)	∑H_2_S(mM)	DOC(mM)	Cu(μM)	Zn(μM)	Ag(μM)	MeHg(μM)	tHg(μM)	MeHg/ tHg %	Fillings/ Surfaces	Day of theWeek
**DWW7**	7.23	28	0.03	0.35	19.3	252	113	28	**2.17E-03**	1.34	0.16	3/6	Tuesday
DWW10	7.97	15	0.01	0.22	23.0	173	75	26	1.90E-04	0.38	0.05	1/3	Friday
DWW11	8.22	6.4	0.58	1.44	31.3	551	199	18	2.39E-04	0.78	0.03	2/5	Monday
**DWW14**	6.92	12	1.46	1.07	14.2	101	49	29	**1.32E-03**	0.38	0.35	1/2	Thursday
**DWW15**	7.30	39	0.51	1.06	26.1	692	245	19	**2.51E-03**	0.67	0.37	2/4	Friday
**DWW16**	7.19	9.4	0.31	1.44	53.4	1086	459	12	**3.99E-03**	0.71	0.56	2/4	Tuesday
**DWW19**	7.81	45	1.39	0.42	2.5	362	199	13	**2.93E-03**	0.77	0.38	2/5	Thursday
**DWW24**	7.21	27	0.74	0.39	6.0	614	214	12	**2.12E-03**	0.85	0.25	2/6	Thursday
DWW25	8.04	76	1.12	0.41	18.1	677	229	14	8.16E-05	0.75	0.01	2/5	Friday
**DWW26**	7.01	32	0.89	1.41	58.9	61	12	12	**1.53E-03**	4.25	0.04	4/9	Monday
DWW29	7.92	64	0.91	0.10	72.5	645	382	17	2.94E-04	0.70	0.04	2/4	Wednesday
DWW33	7.82	69	2.70	2.01	59.0	1023	505	13	1.80E-04	1.38	0.01	2/6	Tuesday
DWW39	7.95	22	BDL	0.31	BDL	409	168	14	4.37E-04	6.10	0.01	3/9	Wednesday
**DWW40**	7.56	24	0.01	0.07	BDL	299	168	16	**1.78E-03**	0.12	1.52	1/2	Thursday

Only geochemical parameters observed >85% of the time in the 14 samples are shown. High MeHg DWW samples denoted by bold font.

Abbreviations: MeHg, Methyl Mercury; tHg, Total Mercury. Fillings = Number of amalgam restorations, removals, or placements during preceding sampling day. Surfaces = total number of exposed surfaces for amalgam restorations, removals, or placements during preceding sampling day. BDL: Below detection limit for this analyte.

**Table 2:** Pearson correlation matrix for geochemical characteristics of the DWW samples by linear regression analyses.

Pearson (*r)*	pH	tHg(μM)	MeHg/tHg %	Hg(HS)_2_(μM)	SO_4_^2−^(mM)	∑H_2_S(mM)	DOC(mM)
MeHg (μM)	−0.64**	−0.18	0.48*	0.13	−0.21	0.08	−0.12
pH	0.00	0.00	−0.27	−0.52*	0.03	−0.21	−0.03
tHg (μM)		0.00	**−0.36**	0.59**	−0.14	0.06	−0.01
MeHg/tHg %			0.00	−0.19	−**0.29**	−0.23	**−0.36**
Hg(HS)_2_ (μ**M**)				0.00	−0.02	0.29	0.31
SO_4_^2−^ (m**M**)					0.00	0.55*	**0.35**
∑H_2_S (mM)						0.00	0.52*

Significant correlation: *P < 0.05; **P < 0.01, Significant Pearson correlations coefficients (*r*) are highlighted.

**Table 3:** Most abundant bacterial families in the 14 DWW sample subset.

DWW7	%	DWW10	%	DWW11	%	DWW14	%	DWW15	%
*Xanthomonadaceae*	90	*Xanthomonadaceae*	29	Uncl. Bacteria	23	Rhodocyclaceae	15	Rhodocyclaceae	17
Uncl. *Enterobacteriaceae*	8	*Pseudomonadaceae*	20	Rhodocyclaceae	19	Cyanobacteria	13	**Desulfobulbaceae**	**17**
Uncl. Bacteria	0.6	Alcaligenaceae	19	**Desulfobulbaceae**	**11**	*Aeromonadaceae*	12	Uncl. Bacteria	16
Uncl. α-proteobacteria	<1	*Enterobacteriaceae*	16	Helicobacteraceae	7	**Desulfobulbaceae**	**11**	**Syntrophobacteraceae**	**10**
Flavobacteriaceae	<1	**Desulfobulbaceae**	**5**	**Desulfovibrionaceae**	**6**	Uncl. Bacteria	10	*Aeromonadaceae*	9
Uncl. Sphingobacteriales	<1	Leptotrichiaceae	4	*Aeromonadaceae*	6	**Syntrophobacteraceae**	**6**	**Desulfovibrionaceae**	**8**
Uncl. γ-proteobacteria	<1	Hydrogenophilaceae	3	Uncl. β-proteobacteria	5	**Desulfovibrionaceae**	**4**	**Uncl. δ-proteobacteria**	**8**
Lactobacillaceae	<1	Uncl. Bacteria	2	**Syntrophobacteraceae**	**3**	Helicobacteraceae	4	Uncl. β-proteobacteria	3
DWW16	%	DWW19	%	DWW24	%	DWW25	%	**DWW26**	%
*Xanthomonadaceae*	53	Rhodocyclaceae	35	*Staphylococcaceae*	73	*Pseudomonadaceae*	42	*Aeromonadaceae*	16
Flavobacteriaceae	13	Uncl. Bacteria	13	*Xanthomonadaceae*	17	*Xanthomonadaceae*	39	Rhodocyclaceae	11
**Uncl. Desulfovibrionaceae**	**11**	**Desulfobulbaceae**	**10**	Uncl. Bacteria	3	*Enterobacteriaceae*	15	**Desulfobulbaceae**	**10**
Oxalobacteraceae	8	Campylobacteraceae	5	Enterobacteriaceae	3	Campylobacteraceae	2	**Syntrophobacteraceae**	**10**
Uncl. Enterobacteriaceae	5	**Uncl. Desulfobacteraceae**	**4**	Oxalobacteraceae	1	Rhodocyclaceae	1	**Uncl. Syntrophobacteraceae**	**8**
Uncl. Bacteria	4	*Bacillaceae*	4	Comamonadaceae	1	Propionibacteriaceae	0.8	**Uncl. δ-proteobacteria**	**7**
Alcaligenaceae	4	Bradyrhizobiaceae	4	**Uncl. Desulfovibrionaceae**	<**1**	Streptococcaceae	<1	**Desulfovibrionaceae**	**5**
**Desulfovibrionaceae**	**1**	Uncl. β-proteobacteria	3	Uncl. Actinomycetales	<1	**Syntrophobacteraceae**	<**1**	Uncl. Bacteria	4
DWW29	%	DWW33	%	DWW39	%	**DWW40**	%		
Acetobacteraceae	23	Uncl. Bacteria	77	*Xanthomonadaceae*	40	*Pseudomonadaceae*	49		
Carnobacteriaceae	12	Uncl. Proteobacteria	20	Cyanobacteria	16	*Xanthomonadaceae*	34		
Anaerolineaceae	8	*Xanthomonadaceae*	0.8	Uncl. Clostridiales	16	*Enterobacteriaceae*	9		
*Xanthomonadaceae*	7	Flavobacteriaceae	0.6	Uncl. Bacteria	9	Alcaligenaceae	5		
Comamonadaceae	6	*Burkholderiales*	0.5	*Staphylococcaceae*	9	Uncl. Actinomycetales	1		
Leptotrichiaceae	6	Enterobacteriaceae	0.4	Alcaligenaceae	3	Uncl. Corynebacterineae	<1		
*Streptococcaceae*	6	Cyanobacteria	<1	Flavobacteriaceae	2	Uncl. Bacteria	<1		
Moraxellaceae	5	**Uncl. δ-proteobacteria**	<**1**	Uncl. Firmicutes	1	Uncl. Xanthomonadaceae	<1		

Bacterial composition is given at family level or at higher taxonomic level if the unclassified sequence could not be assigned to family or genus. High MeHg DWW samples as well as deltaproteobacteria are noted in bold font. Bacterial families known to have Hg resistance are shown in *italics*. Proteobacteria sub-divisions are denoted by the Greek symbols α, β, γ, δ, ε. Uncl: Unclassified.

**Table 4:**
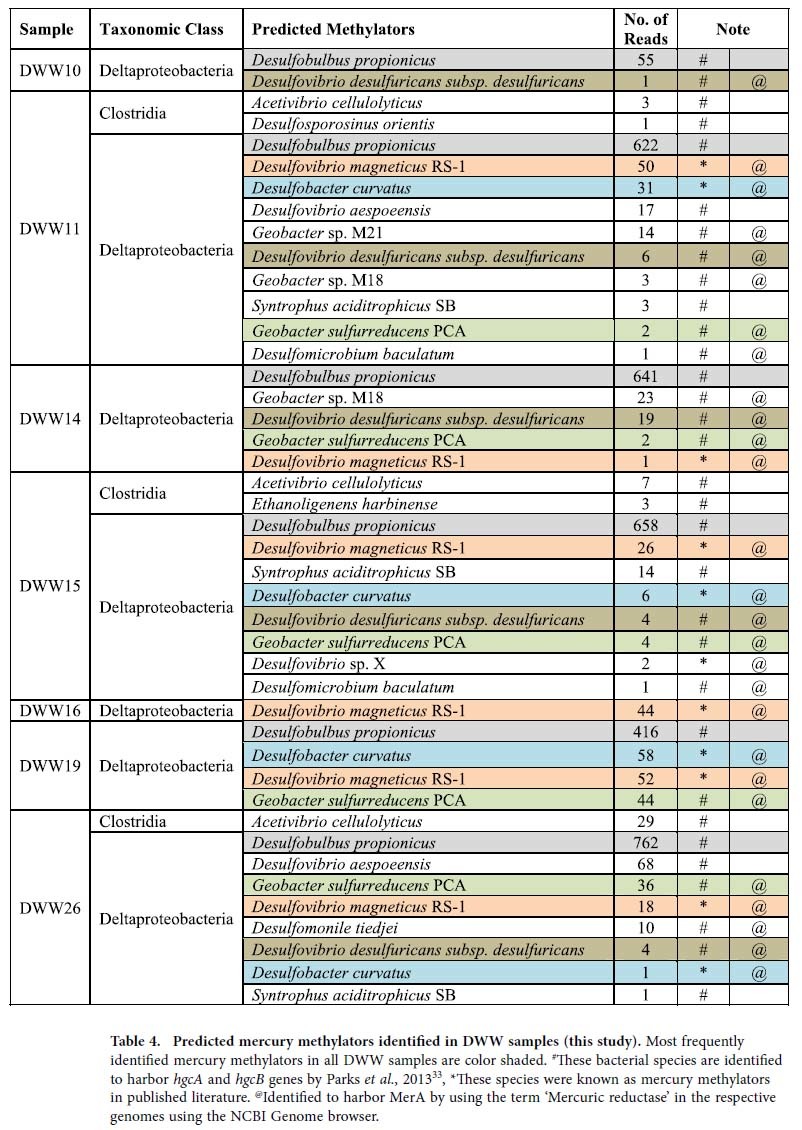
Predicted mercury methylators identified in DWW samples (this study).

**Table 5:** Correlations between the relative abundances of the microbial diversity at dominant bacterial taxa and DWW characteristics by linear regression analyses.

Phylum	MeHg(μM)	pH	tHg(μM)	MeHg/tHg %	Hg(HS)_2_(μM)	SO_4_^2−^(mM)	∑H_2_S(mM)	DOC(mM)
*Actinobacteria*	−0.05	−0.50*	0.03	0.25	0.48*	0.15	0.05	0.03
*Bacteroidetes*	**0.44**	−0.18	−0.04	0.08	−0.03	−0.13	**0.30**	0.49*
*Chloroflexi*	−0.13	0.23	−0.12	−0.18	−0.06	0.13	−0.22	0.49*
*Cyanobacteria*	−0.23	−0.07	0.53*	−0.13	−0.01	−0.04	−0.10	**−0.31**
*Proteobacteria*	0.22	−0.03	−0.26	**0.34**	0.01	−0.58*	**−0.40**	−0.27
*Synergistetes*	0.03	**−0.45**	0.23	−0.14	0.69*	0.10	0.49*	0.24
Unclassified bacteria	−0.23	0.17	0.03	−0.22	−0.08	0.74**	0.72**	**0.38**
Class								
*Alphaproteobacteria*	**−0.25**	0.24	−0.13	−0.18	−0.14	0.13	**−0.26**	0.54*
*Betaproteobacteria*	0.07	0.16	−0.23	−0.14	−0.13	**0.40**	**0.39**	0.07
*Deltaproteobacteria*	**0.28**	**−0.39**	0.04	−0.06	**0.45**	0.15	0.53*	0.15
*Gammaproteobacteria*	−0.08	0.04	0.14	0.18	−0.13	**−0.32**	**−0.39**	**−0.31**
Genus								
*Desulfobulbus*	−0.13	0.12	−0.23	−0.17	−0.05	−0.09	0.02	−0.13
*Desulfovibrio*	0.18	−0.17	−0.09	−0.06	0.18	0.07	0.55*	0.10
*Desulfuromonas*	−0.05	**−0.31**	−0.23	0.01	−0.11	0.20	**0.29**	−0.13
*Geobacter*	0.13	0.23	0.01	−0.11	0.14	0.12	0.25	−0.05
Unclassified Deltaproteobacteria	0.56*	**−0.33**	**−0.27**	0.71**	−0.10	**−0.27**	−0.14	−0.22

Pearson’s correlations coefficients (*r*) are shown for each taxon with significant correlation highlighted. *P < 0.05; **P < 0.01, significant correlation, *P*-values at 0.1 are highlighted in bold.

**Table 6:** SIMPER analysis identifies top abundant taxa that contributed most of the dissimilarities (≥1%) between the microbial communities from high and low MeHg samples.

Phylum: High MeHg *vs.* Low MeHg Overall average dissimilarity 41.6%				
Phylum^a^	Average dissimilarity^c^	Contribution %^d^	Mean abundance High MeHg (%)^e^	Mean abundance Low MeHg (%)
*Proteobacteria*	16.7	39.5	73.2	59.4
Unclassified	10.8	25.4	7.6	21.8
*Firmicutes*	8.6	20.3	12.2	10.2
*Cyanobacteria*	2.0	4.7	1.6	3.0
*Bacteroidetes*	1.3	3.0	2.1	1.6
*Chloroflexi*	0.9	2.1	0.7	1.5
*Fusobacteria*	0.8	1.9	0.1	1.6
*Actinobacteria*	0.5	1.3	1.1	0.4
Class: High MeHg vs. Low MeHg Overall average dissimilarity 42.7%				
Class^b^	Average dissimilarity	Contribution %	Mean abundance High MeHg (%)	Mean abundance Low MeHg (%)
*Gammaproteobacteria*	19.2	42.9	53.9	61.3
*Deltaproteobacteria*	10.7	23.9	22.3	7.2
*Betaproteobacteria*	8.1	18.1	19.8	19.4
*Alphaproteobacteria*	4.9	11.1	1.4	9.9
*Epsilonproteobacteria*	1.8	4.0	2.5	2.2

^a^Phylum level for Bacteria,

^b^Class level for Proteobacteria are displayed,

^c^Average dissimilarity among the taxa,

^d^Contribution of each taxon to the overall dissimilarity between the high and low MeHg groups,

^e^Mean abundance of each OTU.

**Table 7:**
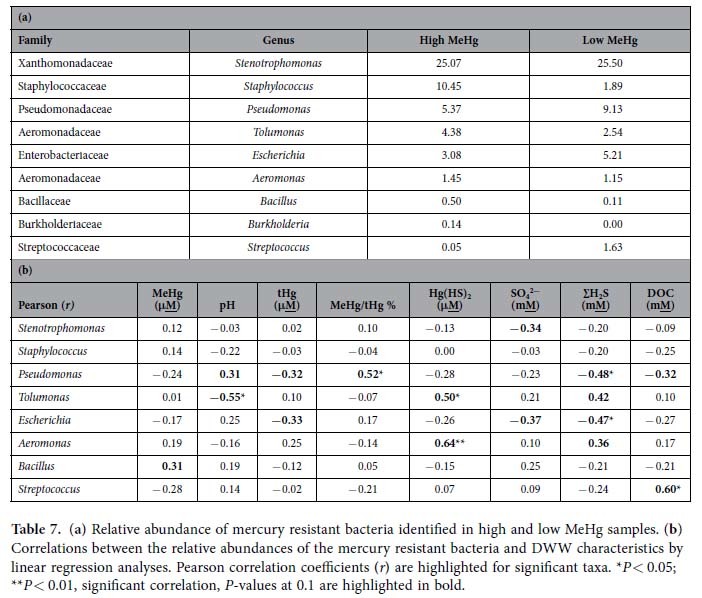


**Table 8:** Richness and diversity measures in the 14 DWW sample subset including number of OTUs, observed and estimated species richness.

Sample	Richness Estimator			Diversity Estimators				
	S_obs_^a^	Chao1^b^	ACE	Shannon *H*	Simpson	Evenness	% Coverage	% Inv. Comp.^c^
**DWW7**	24	37	36	1.0	0.46	0.3	98	65
DWW10	28	34	46	2.0	0.16	0.6	97	82
DWW11	808	632	1360	4.8	0.03	0.7	88	128
**DWW14**	274	398	418	3.9	0.04	0.7	95	69
**DWW15**	422	354	720	4.2	0.04	0.7	89	119
**DWW16**	43	111	92	1.7	0.30	0.5	99	39
**DWW19**	133	179	134	3.3	0.05	0.7	97	75
**DWW24**	31	44	58	1.1	0.55	0.3	98	70
DWW25	59	60	94	1.6	0.27	0.4	99	98
**DWW26**	435	473	568	4.5	0.03	0.7	95	92
DWW29	112	89	139	3.1	0.06	0.7	99	126
DWW33	101	56	112	1.9	0.31	0.4	99	180
DWW39	27	34	42	2.1	0.16	0.6	98	79
**DWW40**	49	77	80	1.5	0.31	0.4	99	64

Phylotype richness, diversity and evenness estimates are based on 97% OTU clusters of the 14 DWW samples.

Abbreviations: OTU, Operational taxonomic unit; ACE, abundance-based coverage estimator; High MeHg DWW samples denoted by bold font.

^a^S_obs_, Observed richness,

^b^Chao1 Estimate of OTU richness, (values with 95% confidence interval),

^c^Total observed richness/Chao1 estimate ×100.

% Good’s coverage of library was calculated as: G = [1−(*n/N*)] 100%, where n is the number of OTUs and N is the total number of sequences in the sample.
