# Supplementary material for: Geochemical influences and mercury methylation of a dental wastewater microbiome
Source: Sci Rep. 2015 Aug 14;5:12872. doi: 10.1038/srep12872 (PMC4642505; doi:10.1038/srep12872)
Supplement: Supplementary Information [file srep12872-s1.pdf]

SUPPLEMENTARY DATA

Geochemical influences and mercury methylation of a dental wastewater microbiome

Asha Rani[1], Karl J. Rockne[1*], James Drummond[2], Muntasar Al-Hinai[3], and Ravi Ranjan[4]

[1]Department of Civil and Materials Engineering, University of Illinois, Chicago, IL, USA

[2]Department of Bioengineering, University of Illinois, Chicago, IL, USA

[3]Department of Orthodontics, University of Illinois, Chicago, IL, USA

[4]Department of Medicine, University of Illinois, Chicago, IL, USA

***Corresponding Author**

Dr. Karl J. Rockne, Ph.D., P.E.

Department of Civil and Materials Engineering

University of Illinois at Chicago

2093 Engineering Research Facility, M/C 246

842 West Taylor St, Chicago, IL 60607-7023

Phone: 312-413-0391; Fax: 312-996-2426 E-mail: krockne@uic.edu

SUPPLEMENTARY DATA

Supplementary Methods
Supplementary Results
References
Supplementary Figures S1 to S5
Supplementary Table S1 to S3

SUPPLEMENTARY METHODS

DWW sample collection: The clinic wastewater line is fitted with an inline 45 L sample tank to facilitate sampling. The tank is approximately 50 m from the dental clinic and receives all wastewater from the clinic sinks and dental units. DWW sits in the tank for a period of time depending on clinic usage. The holding tank was completely flushed at 17:00 h every Sunday and samples were collected every day of the week at 17:00 h after the clinic had closed. Every sampling started with a new certified clean container (250 mL Teflon bottle, Nalge Nunc International, Rochester, NY) in which the regular DWW flow was diverted to fill the container, and the fill duration was recorded. DWW samples were preserved with HCl immediately after measuring the pH and stored at -20°C until analysis of solids content, dissolved and total organic carbon, tHg, MeHg, heavy metals and DNA isolation[1].

DWW characterization: DWW samples were thawed, homogenized by vortexing at a high rate for approximately 30 s prior to sub-sampling for parameter measurements. Dissolved and total organic carbon (DOC and TOC) were determined for known volumes of filtered and unfiltered aqueous samples absorbed in chromosorb W/AW (CE Elantech Lakewood, NJ) and subjected to elemental analysis (Flash EA 1112; Thermo-Quest/CE Elantech, Lakewood, NJ) as described previously[2,3]. Anions were measured by ion chromatography (Dionex IC 25, Dionex, Sunnyvale, CA) as described previously[4]. Filtered and unfiltered samples for sulfide were preserved according to Standard Methods[5] and quantified by iodometric titration modified for small sample volumes[3,6].

Mercury analysis: Total mercury was quantified by EPA standard method 1631 and 1630[7,8] using a Brooks-Rand Cold Vapor Atomic Fluorescence Spectrometry (CVAFS) System (model III, Brooks-Rand, Seattle, WA)[1,3]. For inorganic Hg measurement, samples were homogenized, acidified (0.4% 12N HCl), diluted (1:2000 v/v), and processed as for water samples. A 100 mL aliquot from the sample was poured into a fluoropolymer bottle and oxidatively digested at room temperature for 12 h with concentrated BrCl (1% v/v) prepared with KBr, $KBrO_3$ and concentrated HCl (reagent grade, Fisher Scientific, Fairlawn, NJ). Complete destruction of the BrCl was ensured by disappearance of the yellow color. After oxidation, samples were sequentially reduced with $NH_2OH{\cdot}HCl$ (Sigma-Aldrich, St. Louis, MO) to destroy free halogens, and then reduced with $SnCl_2$ (Sigma-Aldrich, St. Louis, MO) to convert Hg^{2+} to volatile Hg^0. For the blanks; 100 mL of

deionized (DI) water was added to the bubbler bottles (rinsed and cleaned with 0.5 mL of $NH_2OH{\cdot}HCl$, 0.5 mL of $SnCl_2$ and connected to flow of N_2 gas for 15 min). The Hg^0 was separated from solution by purging with N_2 and collecting onto a gold trap (Brooks-Rand, Seattle, WA). The analyte was then thermally desorbed from the gold trap into AR gas to the CVAFS cell for detection. The quality was assured through calibration and testing of the oxidation, purging and detection system[3,9].

For MeHg analysis, 45 mL of the diluted sample (up to 1:1000 mL DI) was placed in a fluoropolymer distillation vessel (Brooks-Rand, Seattle, WA) and distilled with 200 µL of 1% ammonium pyrrolidine dithiocarbamate (Alfa Aesar, Ward Hill, MA) at 125°C. The distillate (35 mL) was collected in the receiving vessel under N_2 gas flow. The samples were adjusted to pH 4.9 with an acetate buffer (2 M sodium acetate and 2M glacial acetic acid in DI water; Sigma-Aldrich, St. Louis, MO) and ethylated in a closed bubbler by adding sodium tetraethyl borate (Strem Chemical, Newburyport, MA). The methyl ethyl Hg product was separated from the solution by bubbling with N_2 gas onto a graphitic carbon trap (Carbotrap®; Brooks-Rand, Seattle, WA) and thermally desorbed into AR gas to the separation column followed by pyrolytic decomposition to convert organo-Hg forms to Hg^0, and finally to the CVAFS cell for detection. Quality was ensured through calibration and testing of the distillation, ethylation, purging, and detection systems. Method detection limits (pre-dilution) were approximately 5 nM for MeHg and 30 nM for inorganic Hg[1,3].

Equilibrium speciation modeling: Equilibrium speciation modeling was performed using the program Visual MINTEQ (v. 3.0, KTH Royal Institute of Technology, Stockholm, Sweden), a visual basic version of MINTEQA2 (v. 4.0, CEAM, USEPA, Washington, DC) as described previously[1,3]. Briefly, all chemical data and the pH were input to the model and the main Hg species were reported in the presence of amalgam as described previously[1,3]. DOC-Hg species were also computed using the NICA-Donnan DOC model, although only the primary Hg-sulfide species are reported here as they were dominant[1,3].

Automated ribosomal intergenic spacer analysis (ARISA) of DWW bacterial community: This community fingerprinting method involves PCR amplification from total bacterial community DNA of the intergenic spacer region between 16S SSU and 23S LSU rRNA genes, using

previously published primers, S-D-Bact-1522-b-S-20 and L-D-Bact-132-a-A-18[10-12]. The 16S-23S intergenic spacer region displays significant heterogeneity in length and nucleotide sequence and these variations have been used extensively to distinguish closely-related bacterial species. PCR reactions were performed in 50 μL volumes containing 5 μL of 10× DreamTaq Green Buffer (Fermentas, Glen Burnie, MD), 0.5 μM of each primer, 200 μM of each dNTP (Fermentas, Glen Burnie, MD), 10-15 ng extracted DNA and 1.25 U of DreamTaq Green DNA polymerase. Thermocycling conditions were as follows: initial denaturation at 95°C for 5 min, followed by 28 cycles of denaturing at 94°C for 30 seconds, annealing at 50°C for 1 min, and extension at 72°C for 1 min. Cycling was completed by a final extension at 72°C for 10 min. PCR products were purified with a MoBio UltraClean PCR Clean-up Kit (MoBio Laboratories, Carlsbad, CA) following manufacturer's protocol. Approximate DNA concentrations were assessed using a Nanodrop-2000 spectrophotometer (Thermo Scientific, Wilmington, DE) and normalized to 100 ngμL^{-1} in each DWW sample. PCR products were separated and analyzed on an Agilent 2100 Bioanalyzer using the Agilent DNA 7500 kit (Agilent Technologies, Santa Clara, CA, USA) at the Research Resource Center, DNA Research Facility, University of Illinois, Chicago. Fragment peaks were assigned a size and DNA molarity value using Agilent 2100 Expert Software algorithms. Sample profiles were normalized and log (X+1) transformed in the software package Primer-E[13]. The obtained matrix was further analyzed to determine similarities of the ARISA fingerprints between samples. Cluster analysis and principal coordinates analysis plots were generated from a Bray-Curtis similarity matrix[14] using PAST software v1.82b (http://folk.uio.no/ohammer/past/)[15].

16S rRNA gene tag sequencing and data analysis: The sequencing reactions utilized a Roche 454 FLX platform (Roche, Indianapolis, IN) with titanium reagents and procedures. Additionally, we used a single 35 cycle PCR step with Qiagen HotStar Master Mix and addition of 0.5 U of HotStar HiFidelity Polymerase in each reaction (Qiagen Inc., Valencia, CA). Amplicon sequencing was performed based upon the RTL protocols (as longer average read lengths are generated by the titanium methodology). After sequencing, all failed low-quality sequences and tags were removed as described previously[16]. Raw data from bTEFAP was screened and trimmed based upon quality scores, Phred20 average, and binned into individual sample collections. Sequences were trimmed to 300 bp and sequence reads shorter than this were removed from further analysis. Thereby

ensuring that all sequences evaluated contained sufficient discriminating data from the V1–V3 variable regions of the 16S rRNA gene. Individual collections of sequences were then denoised and assembled into clusters depleted of chimeras using the black box chimera check software B2C2[17]. Final sequences used for analysis had a read length range of 350–450 bp. Bacterial species were identified using RDP classifier and the percentage of each bacterial species were analyzed among the samples based upon normalized numbers of sequence reads (lowest read sample). Because the use of relative abundance can introduce false associations in the data and influence the CA and PCoA, normalized data were used to determine the relative abundance of bacteria at each taxonomic level.

Estimates of microbial diversity: The normalized sequence set of each DWW sample was also individually aligned using the bacteria-alignment model of the RDP. By applying the Complete-Linkage Clustering (or farthest neighbor) sequences in each sample were assigned to OTU/phylotype clusters at 97% cutoff level [complete method available at the RDP Pyrosequencing Pipeline][18,19]. The taxonomy of the sequences was assigned using RDP Bayesian classifier algorithm and a bootstrap value of 80% was used as the default parameter[18,19]. On the basis of these clusters, rarefaction curves, ACE and Chao1 richness were calculated using Rarefaction and Chao1 Estimator. Good's coverage was calculated as $G = 1 - n/N$, where n is the number of phylotypes and N is the total number of sequences in the sample[20].

Cluster Analysis (CA) and Principal Coordinate's Analysis (PCoA): CA and PCoA were also conducted using a matrix of the RDP taxa at each level from phylum to genus and their abundances in each sample. ARISA based CA and PCoA were also performed as described above. Partial least squares-discriminant analysis (PLS-DA) plotting and fold change (FC) analysis of samples based on high and low MeHg groups was performed using METAGENassist[21]. FC analysis was performed to compare the absolute bacterial taxa abundance change between the high and low MeHg group means. FC is calculated as the ratio between group means using data normalization by autoscaling (mean-centered and divided by the standard deviation of each variable). The FC values are log transformed in order for both up and down regulated bacterial taxa to appear symmetrically.

Molecular phylogenetic analysis: Phylogenetic relationships among species were inferred based on the representative sequence for each OTU[22]. To these we added 19 additional known Hg methylating bacteria that have been tested for Hg methylation ability and for which 16S rRNA gene sequences were available in databases[23]. *Desulfobacter postgatei* was used as the reference and outgroup taxon and type strains are indicated by the letter – 'T'. All reference sequences were available from RDP and The National Center for Biotechnology Information (NCBI). Nucleotide sequences were aligned using ClustalW and a phylogenetic tree was constructed using MEGA version-4[24]. The evolutionary history was inferred using the neighbor-joining method and the evolutionary distances were computed using the maximum composite likelihood method[25]. All positions containing gaps and missing data were eliminated using the complete deletion option and high quality alignments were used for phylogenetic tree reconstructions. The bootstrap consensus tree inferred from 1,000 replicates was taken to represent the evolutionary history of the taxa analyzed[26]. The bootstrap values given at nodes are percentages of 1,000 replicates.

Quality assurance: Hg standards were purchased from Perkin Elmer Pure (Shelton, CT) and High Purity Standard (Charleston, SC). The MeHg standards were obtained from Brooks-Rand (Seattle, WA). These standards were used for preparing the standard curve and standard checks during the analytical runs for comparison and to ensure measurement quality. The overall recovery rate of MeHg for the entire extraction and analysis procedure was optimized to 90% ±15%. All chemical data were compared with known standards and reported to two or three significant figures depending upon the total uncertainty at the 95% CI ($U_{95\%} = \sqrt{2(RSD)^2 + B^2}$) from the precision of duplicates and measured analytical bias[5]. Samples above the highest calibration standards were diluted and samples below the lowest achievable calibration standard are reported as below detection limit (BDL). Quality assurance for metals analysis (except Hg) included matrix spikes (143%) and blank recoveries (all BDL) and the U_{95} for total heavy metals (except Hg) was 24.1%[27]. All statistical analyses and exploratory factor analysis was performed using principle components analysis with Varimax rotation using SYSTAT v.12. The whole dataset was tested for normal distribution using the Shapiro–Wilk test and homogeneity of variances. When the assumption of normal distribution was violated the data were transformed as indicated. Cluster analysis (CA) and principal coordinate's analysis (PCoA) were performed to evaluate which geochemical factors predict changes in microbial community structure and key-parameters affecting composition of

DWW community. Statistical analyses of data were checked by one-way analysis of variance (ANOVA) and differences in mean values were accepted as being statistically significant if $P<0.05$. Sampling and analytical protocols for Hg complied with requirements for NELAP accreditation and included matrix spikes and method blanks.

SUPPLEMENTARY RESULTS

Firmicutes: Firmicutes were identified as the third most abundant phylum after Proteobacteria and unclassified bacteria. The highest numbers of Firmicutes were detected in DWW24 (74% of the total bacteria).

Bacteroidetes: In sample DWW16, the highest (12% of total bacteria) reads of Bacteroidetes were identified, followed by DWW29 with 4.5% of Bacteroidetes. In Bacteroidetes total 19 genera were identified.

Actinobacteria: Actinobacteria constituted <4% of the total bacteria in DWW33 DWW26 and DWW15 sample. A total of 14 family were identified with 14 genera and unclassified Actinomycetales.

Cyanobacteria: Cyanobacterial sequences were present in 8 out of 14 DWW samples analyzed in this study. DWW14 and DWW39 constituted 12 to 15% of Cyanobacterial reads out of the total bacteria. Cyanobacterial sequences were dominated by members that we could not assign below the phylum level and were unclassified bacteria derived from Cyanobacteria. Other cyanobacterial sequences in these samples were related to Nostocales, Prochlorales, and unclassified Chroococcales.

Chloroflexi: Chloroflexi were detected in 6 out of 14 DWW samples. Chloroflexi sequences were also dominated by members that could not be assigned below family level and were unclassified Anaerolineaceae members.

Fusobacteria: Fusobacteria were detected only in 4 out of 14 DWW samples studied. Only family identified in Fusobacteria is Fusobacteriaceae.

Acidobacteria and Synergistetes: Acidobacteria were detected in very low abundance (0.02 to 0.7% of total bacteria), in 6 out of 14 DWW samples. Only identified Acidobacteria were unclassified Halophagaceae and *Geothrix*. Synergistetes were identified in 5 DWW samples in low abundance (0.04 to 2%). They were detected as unclassified Synergistaceae.

Other Phyla: The remaining 16 phyla were grouped as other phyla, which were detected at very low abundance (0.02 to 1.3%) in 10 out of 14 DWW samples. These phyla were identified as Spirochaetes, TM7, Thermotogae, Planctomycetes, OP11, Lentisphaerae, Archaea, Verrucomicrobia, Tenericutes, BRC1, Deferribacteres, Gemmatimonadetes, OD1, WS3, SR1 and unclassified roots. Candidate phyla TM7, OP11, BRC1, OD1, WS3 and SR1 were represented by only a few sequences (<30).

Unclassified Bacteria: More than 9900 reads remained as unclassified bacteria and accounted for up to 96% of the total bacteria in DWW samples. Highest levels of unclassified bacteria were detected in DWW33, DWW15 and DWW11.

REFERENCES

1 Zhao, X. *Methyl mercury in dental wastewater*. PhD Thesis, University of Illinois, (2009).

2 Buckley, D. R., Rockne, K. J., Li, A. & Mills, W. J. Soot deposition in the Great Lakes: implications for semi-volatile hydrophobic organic pollutant deposition. *Environ. Sci. Technol.* **38**, 1732-1739 (2004).

3 Zhao, X. *et al.* Characterization of methyl mercury in dental wastewater and correlation with sulfate-reducing bacterial DNA. *Environ. Sci. Technol.* **42**, 2780-2786 (2008).

4 Rockne, K. & Brezonik, P. Nutrient Removal in a Cold-Region Wastewater Stabilization Pond: Importance of Ammonia Volatilization. *J. Environ. Eng.* **132**, 451-459 (2006).

5 Clesceri, L., Greenberg, A. & Eaton, A. *Standard Methods for the Examination of water and Wastewater*. 21st edn, (American Public Health Association, American Water Works Association, and Water Environment Federation, 2005).

6 Rockne, K. J. & Strand, S. E. Biodegradation of bicyclic and polycyclic aromatic hydrocarbons in anaerobic enrichments. *Environ. Sci. Technol.* **32**, 3962-3967 (1998).

7 USEPA. Method 1630: Methyl mercury in water by distillation, aqueous ethylation, purge and trap, and CVAFS 01A0007846. (2001).

8 USEPA. Method 1631: Mercury in water by oxidation, purge and trap, and cold vapor atomic fluorescence spectrometry EPA 821/R-96-012. (2002).

9 Zhao, X., Rockne, K. J. & Drummond, J. L. Aeration prevents methyl mercury production in dental wastewater. *Journal of Environmental Science and Health, Part A* **47**, 598-604 (2012).

10 Lane, D. J. 16S/23S rRNA sequencing. *Nucleic Acid Techniques in Bacterial Systematics*, 125-175 (1991).

11 Cardinale, M. *et al.* Comparison of different primer sets for use in automated ribosomal intergenic spacer analysis of complex bacterial communities. *Appl. Environ. Microbiol.* **70**, 6147-6156 (2004).

12 Kostka, J. E. *et al.* Hydrocarbon-degrading bacteria and the bacterial community response in gulf of Mexico beach sands impacted by the deepwater horizon oil spill. *Appl. Environ. Microbiol.* **77**, 7962-7974 (2011).

13 Clarke KR, R. W. *Change in marine communities: an approach to statistical analysis and interpretation.* 2nd edn, (PRIMER-E Ltd, 2001).

14 Bray, J. R. & Curtis, J. T. An ordination of the upland forest communities of southern Wisconsin. *Ecol. Monogr.* **27**, 325-349 (1957).

15 Hammer O, H. D., Ryan PD. PAST: Paleontological statistics software package for education and data analysis. *Palaeontologia Electronica* **4**, 9 (2001).

16 Acosta-Martínez, V., Dowd, S., Sun, Y. & Allen, V. Tag-encoded pyrosequencing analysis of bacterial diversity in a single soil type as affected by management and land use. *Soil Biol. Biochem.* **40**, 2762-2770 (2008).

17 Gontcharova, V. *et al.* Black Box Chimera Check (B2C2): a windows-based software for batch depletion of chimeras from bacterial 16S rRNA gene datasets. *Open Microbiol. J.* **4**, 47-52 (2010).

18 Wang, Q., Garrity, G. M., Tiedje, J. M. & Cole, J. R. Naive Bayesian classifier for rapid assignment of rRNA sequences into the new bacterial taxonomy. *Appl. Environ. Microbiol.* **73**, 5261-5267 (2007).

19 Cole, J. R. *et al.* The Ribosomal Database Project: improved alignments and new tools for rRNA analysis. *Nucleic Acids Res.* **37**, D141-145 (2009).

20 Good, I. J. The population frequencies of species and the estimation of population parameters. *Biometrika* **40**, 237-264 (1953).

21 Arndt, D. *et al.* METAGENassist: a comprehensive web server for comparative metagenomics. *Nucleic Acids Res.* **40**, W88-W95 (2012).

22 Schloss, P. D., Gevers, D. & Westcott, S. L. Reducing the effects of PCR amplification and sequencing artifacts on 16S rRNA-based studies. *PLoS One* **6**, e27310 (2011).

23 Gilmour, C. C. *et al.* Sulfate-reducing bacterium *Desulfovibrio desulfuricans* ND132 as a model for understanding bacterial mercury methylation. *Appl. Environ. Microbiol.* **77**, 3938-3951 (2011).

24 Tamura, K., Dudley, J., Nei, M. & Kumar, S. MEGA4: Molecular Evolutionary Genetics Analysis (MEGA) Software Version 4.0. *Mol. Biol. Evol.* **24**, 1596-1599 (2007).

25 Tamura, K. & Nei, M. Estimation of the number of nucleotide substitutions in the control region of mitochondrial DNA in humans and chimpanzees. *Mol. Biol. Evol.* **10**, 512-526 (1993).

26 Felsenstein, J. Confidence limits on phylogenies: an approach using the bootstrap. *Evolution* **39**, 783-791 (1985).

27 Rockne, K., Kaliappan, R. & Bourgon, G. Sediment gas ebullition study, Grand Calumet River, Western Branch, Reaches 1 and 2. 234 (2011).

SUPPLEMENTARY FIGURE AND TABLE LEGENDS

Supplementary Figure Legends

Supplementary Figure S1. **(a)** Correlation of MeHg and sulfide levels, (**b)** pH and sulfide levels detected in high and low MeHg samples. High MeHg samples are shown by filled black circles and low MeHg samples are shown by open circle symbols. Note that all samples with low MeHg samples were at pH >7.8. **(c-d)** Box plot for sulfate and sulfide levels in high and low MeHg samples. There was no statistical significant difference in levels of sulfate and sulfide among the groups. **(e)** pH values in high and low MeHg group. pH was significantly different among the groups (Student *t*-test, $P < 0.01$).

Supplementary Figure S2. Box plots showing mean concentrations of (a) Ag, **(b)** Cu, **(c)** MeHg and **(d)** tHg as a function of day of the week, and mean **(e)** Ag, **(f)** Cu, **(g)** MeHg and **(h)** tHg as a function of number of fillings in the clinic. The horizontal line is the mean and the boxes represent the 25%/75% and the fences represent the 5%/95% confidence intervals for the mean. In general, Ag and Cu did not vary significantly with day of week or with number of fillings, while tHg was a strong function of number of fillings. Both MeHg and tHg exhibited significant differences with the day of the week.

Supplementary Figure S3. Data normalization view. Box plots and kernel density plots show the distribution of bacterial taxa identified in DWW samples before (left) and after (right) normalization. Auto scaling is mean-centered and divided by the standard deviation of each variable.

Supplementary Figure S4. Microbial diversity index values in 14 DWW samples as estimated by pyrosequencing and ARISA as a function of total OTUs observed. Shown are **(a)** Shannon diversity index versus pyrosequencing OTUs, **(b)** Shannon diversity versus ARISA OTUs, **(c)** log-transformed Shannon index, Chao1 estimate of OTU richness, and species evenness and ACE index data versus pyrosequencing OTUs. Note the similar pattern and high association between observed diversity with species richness as estimated by both pyrosequencing and ARISA analyses.

Supplementary Figure S5. Shown are **(a)** a comparison of OTUs (y1 axis, log scale) and Shannon diversity indices (y2 axis) observed from pyrosequencing and ARISA analyses of selected DWW samples, **(b)** rarefaction curves of 14 DWW samples using 16S rRNA pyrosequencing. 16S rRNA gene sequences were grouped in to same OTUs/phylotypes. The rarefaction curve, plotting the number of observed OTUs as a function of the number of sequences, was computed using RDP Pyrosequencing Pipeline Rarefaction tool.

Supplementary Table Legends

Supplementary Table S1. Descriptive statistics for geochemical parameters in all DWW samples. Statistics are shown only for geochemical parameters that were above the detection limit >65% of the time.

Supplementary Table S2. Summary statistics for Mantel tests. The Mantel statistic *r*(AB) estimates the correlation between two proximity matrices, A and B.

Supplementary Table S3. Oligonucleotides and barcode sequences used in the study.

Supplementary Figure S1

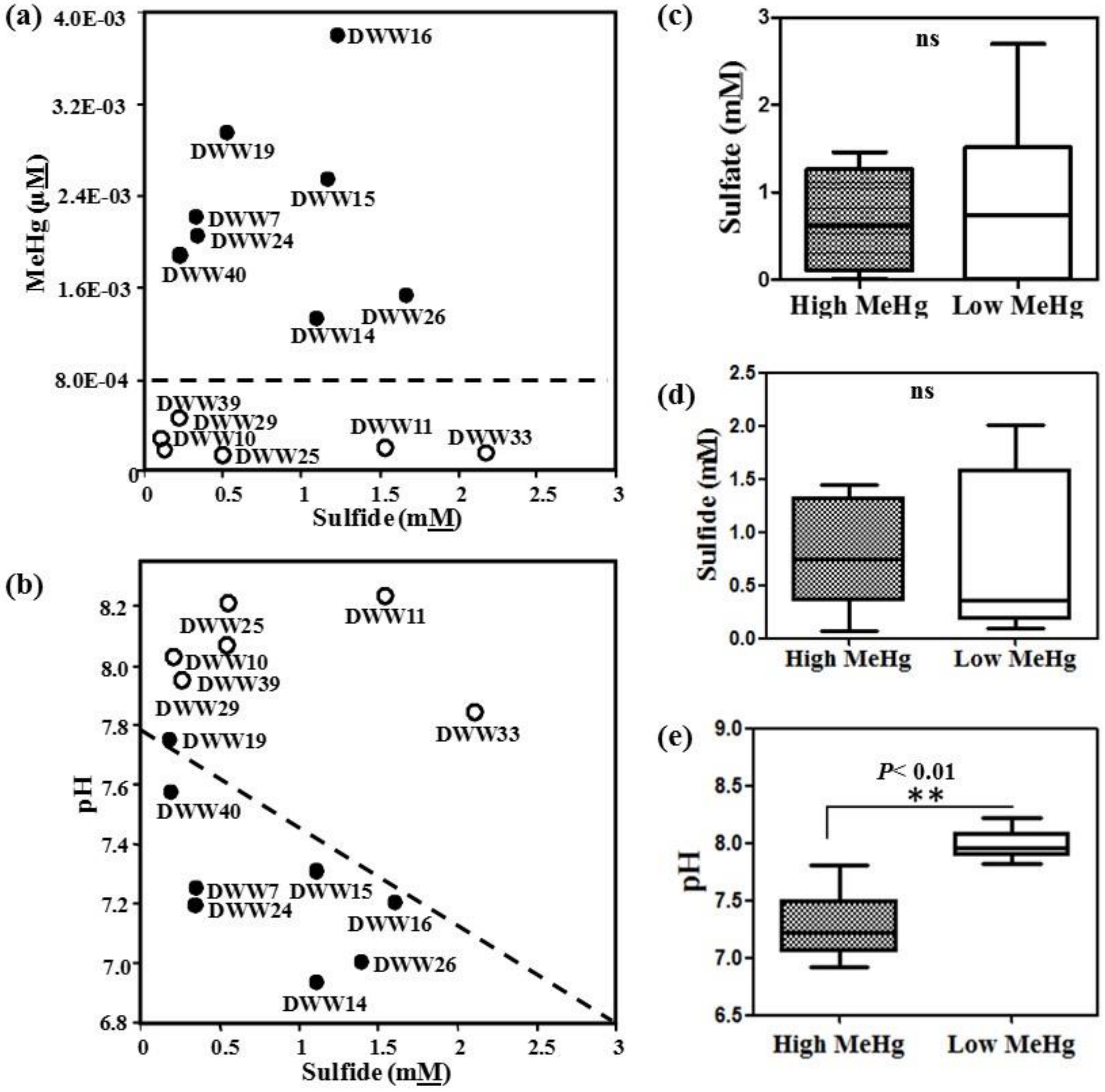

Supplementary Figure S2

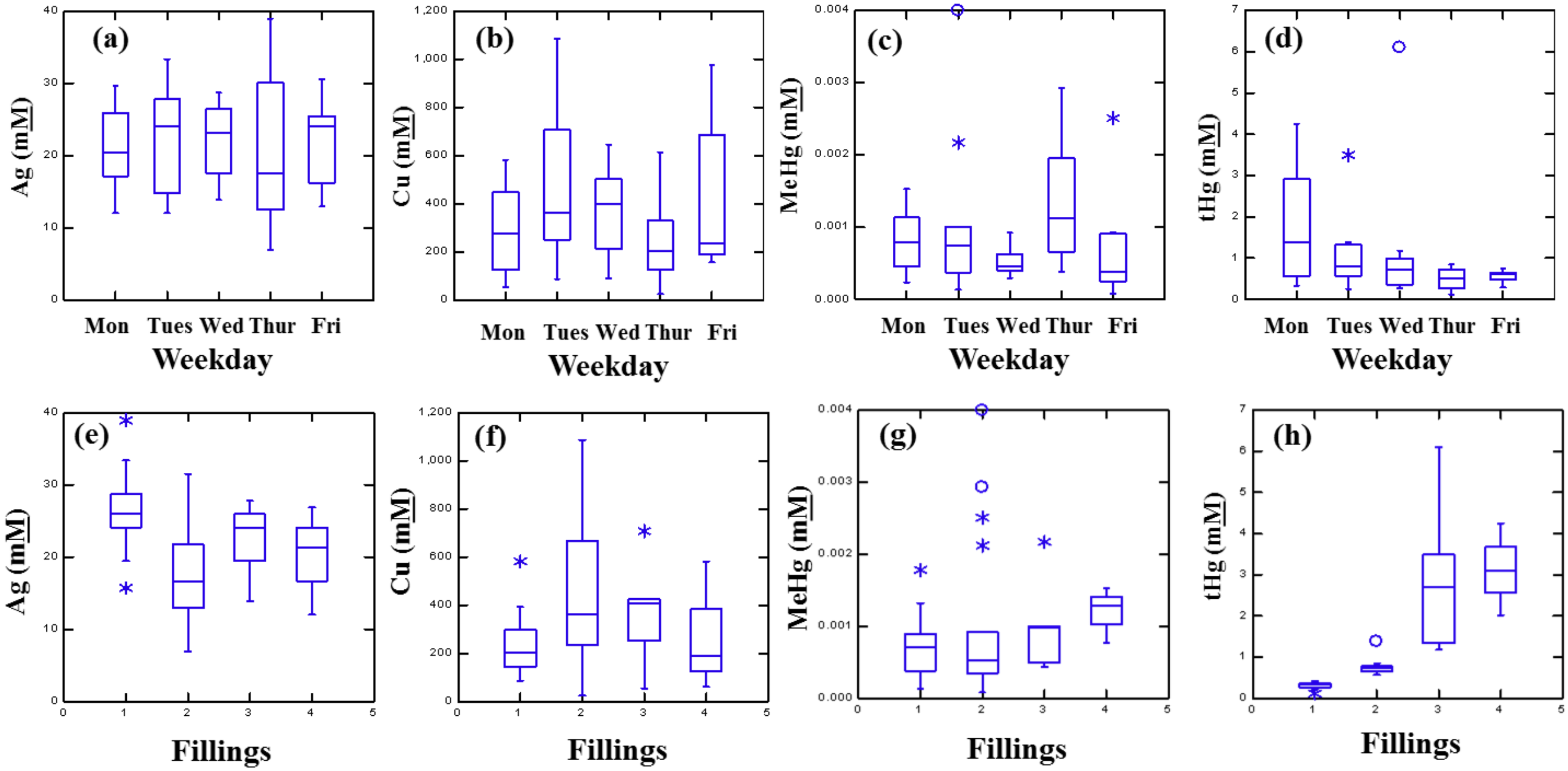

Supplementary Figure S3

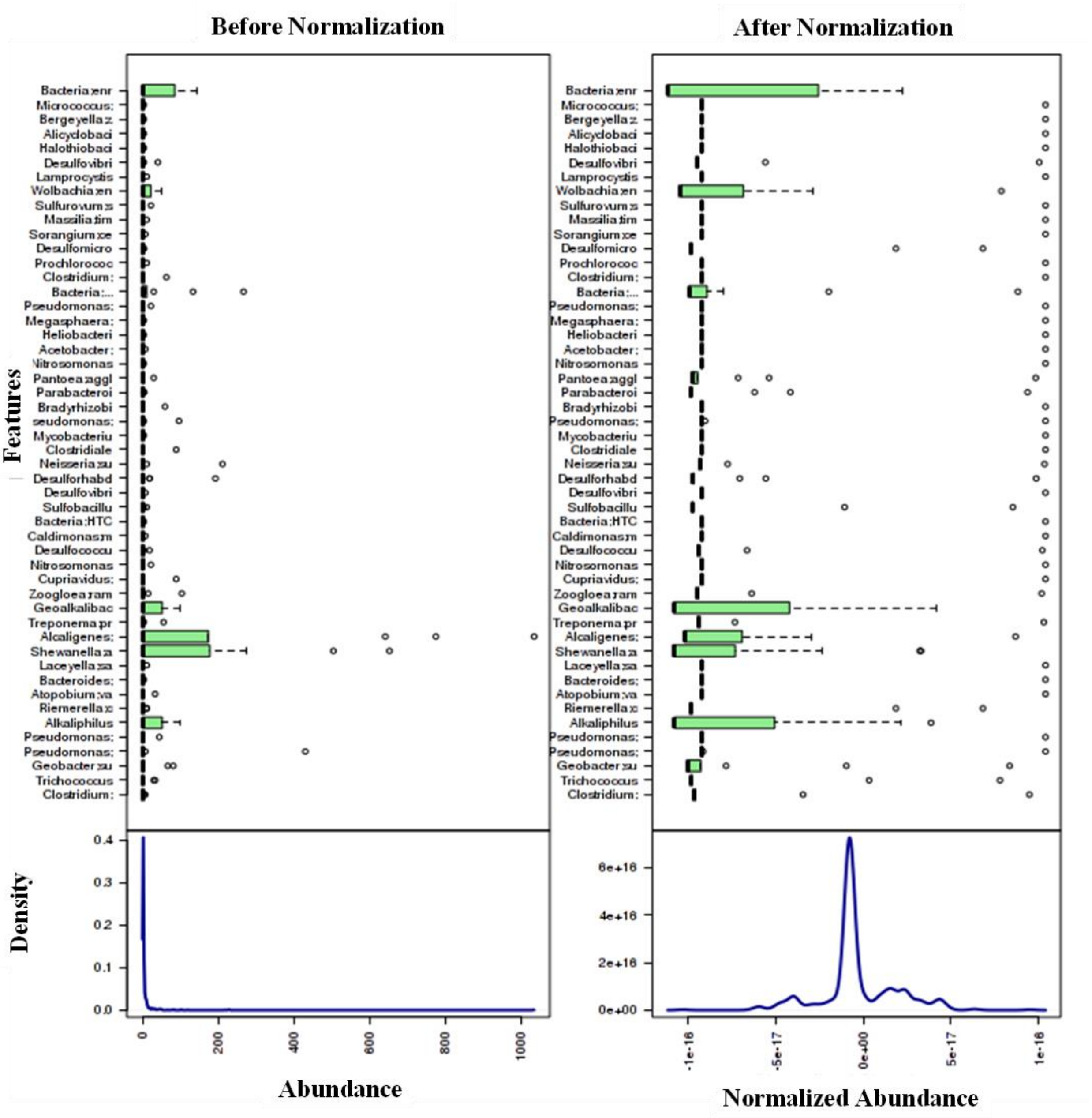

Supplementary Figure S4

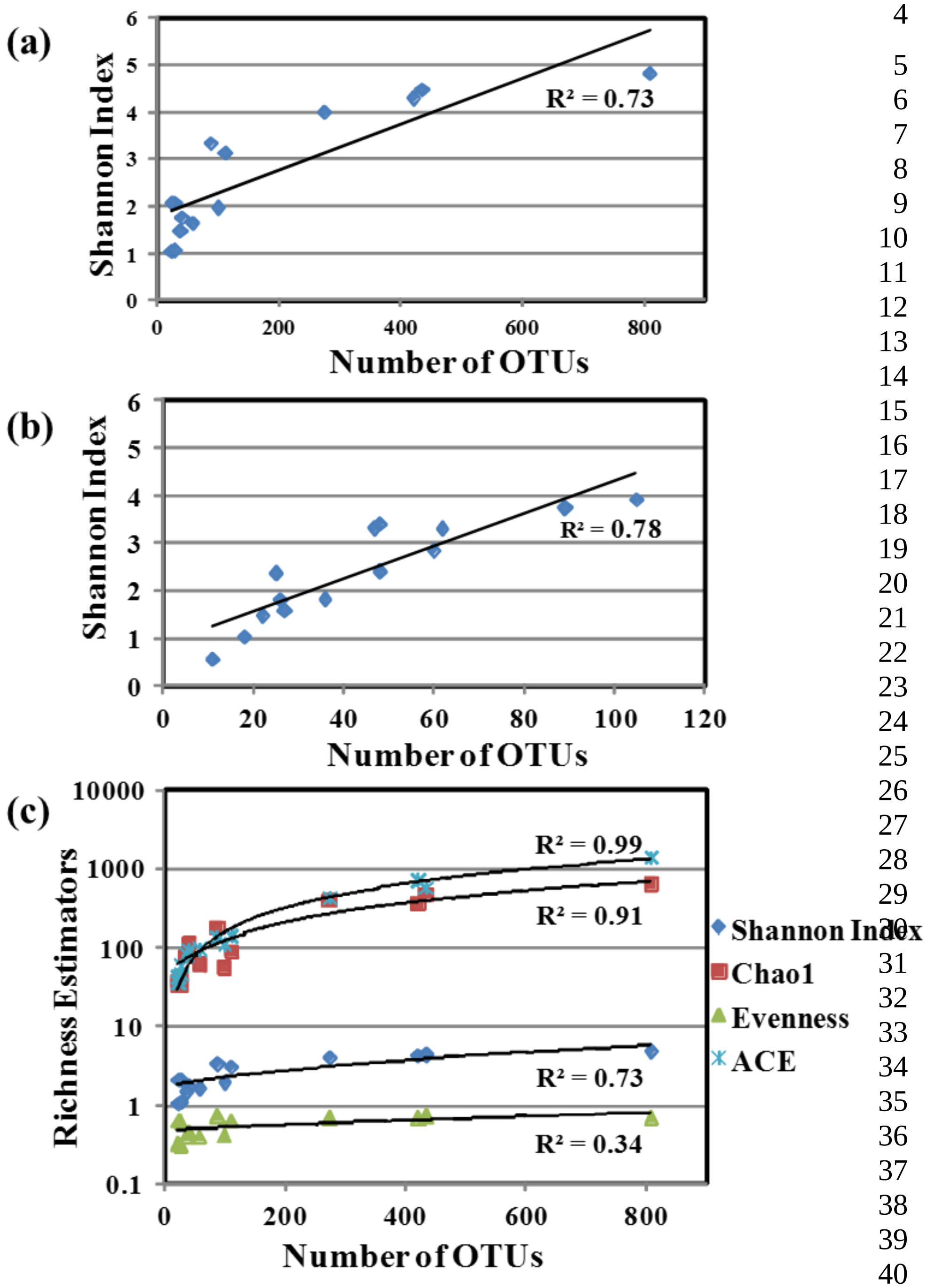

Supplementary Figure S5

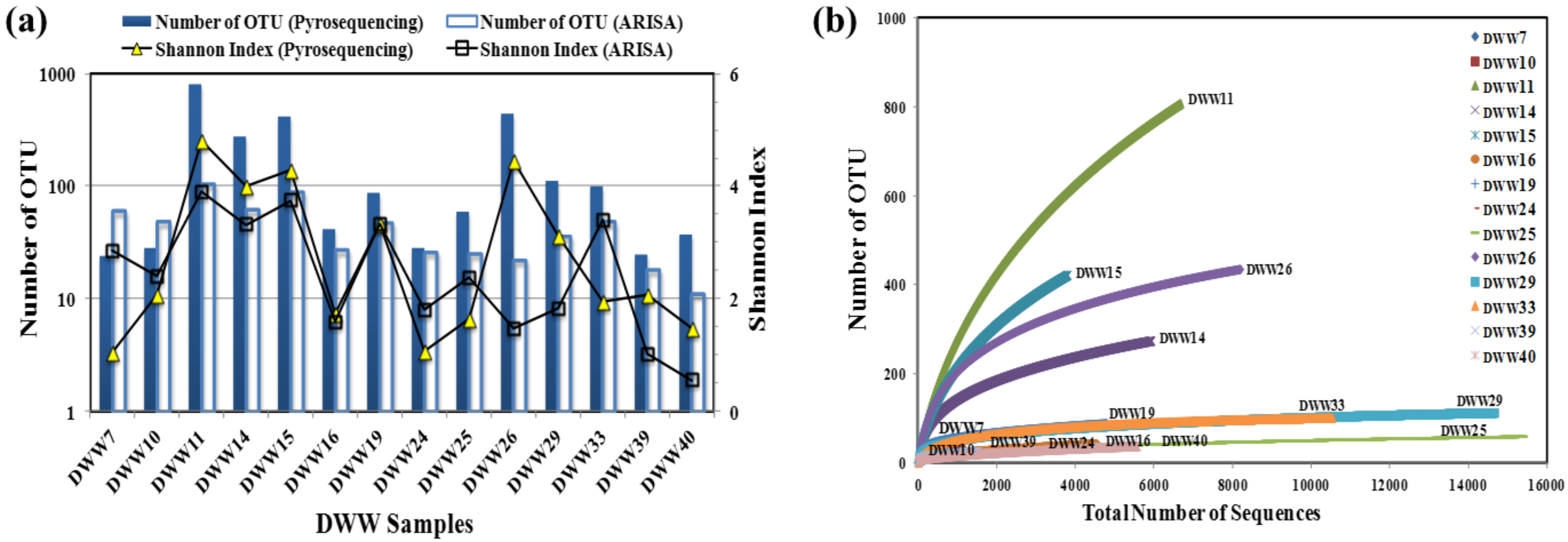

Supplementary Table S1. Descriptive statistics for geochemical parameters in all DWW samples. Statistics are shown only for geochemical parameters that were above the detection limit >65% of the time.

Statistic	pH	F^- (mM)	Br^- (mM)	Cl^- (mM)	NO_2^- (mM)	SO_4^{2-} (mM)	$\sum H_2S$ (mM)	DOC (mM)	Cu (µM)	Zn (µM)	Ag (µM)	MeHg (µM)	tHg (µM)	MeHg/ tHg %
N of cases	40	40	40	40	40	40	40	40	40	40	40	40	40	40
Minimum	5.72	0.00	0.00	6.41	0.00	0.00	0.07	0.00	25.18	12.39	7.0	8.2E-05	0.12	0.007
Maximum	8.31	3.87	0.19	76.1	0.024	2.70	2.01	261	1086	505	38.9	4.0E-03	6.10	1.52
Range	2.60	3.87	0.19	69.7	0.024	2.70	1.94	261	1061	492	32.0	3.9E-03	5.98	1.52
Median	6.98	0.007	0.021	25.0	0.003	0.54	1.14	20.9	315	148	21.3	7.9E-04	0.69	0.089
Arithmetic mean	7.10	0.11	0.034	30.66	0.005	0.62	1.07	28.9	376	170	21.4	9.2E-04	1.06	0.17
SEM	0.12	0.10	0.007	3.02	0.001	0.10	0.09	6.90	43.3	19.1	1.15	5.7E-04	0.20	0.04
Coefficient of variation	0.11	5.68	1.26	0.62	1.24	1.01	0.52	1.51	0.73	0.71	0.34	0.90	1.16	1.48
Skewness (G1)	-0.06	6.31	1.95	0.99	1.72	1.24	-0.29	4.04	1.00	1.12	0.17	1.97	2.63	4.13
Kurtosis (G2)	-1.24	39.9	3.87	0.09	2.48	2.01	-1.05	20.8	0.38	0.84	-0.63	4.29	7.30	20.9
Shapiro-Wilk statistic	0.94	0.16	0.76	0.90	0.77	0.87	0.95	0.59	0.90	0.90	0.97	0.79	0.64	0.56
S-W *P*-value	0.04	0.00	0.00	0.00	0.00	0.00	0.06	0.00	0.00	0.00	0.30	0.00	0.00	0.00
Anderson-Darling statistic	0.67	14.4	3.14	1.45	3.26	1.25	0.77	3.68	1.305	1.19	0.57	2.73	5.44	4.65
A-D *P*-value	0.062	<0.01	<0.01	<0.01	<0.01	<0.01	0.041	<0.01	<0.01	<0.01	0.13	<0.01	<0.01	<0.01

Supplementary Table S2. Summary statistics for Mantel tests. The Mantel statistic *r*(AB) estimates the correlation between two proximity matrices, A and B.

Matrix A[a]	Matrix B[b]	*r* (AB)	*P*
Phylum level	Environmental factors	−0.12	0.24
Class level	Environmental factors	−0.13	0.19
Genus level	Environmental factors	−0.25	0.14
Mercury resistant taxa[c]	Environmental factors	0.16	0.12

Proteobacteria class	Environmental factors	*r* (AB)	*P*
	No. of OTU	**0.38**	**0.06**
	MeHg	**0.25**	**0.12**
	tHg	**0.21**	**0.13**
	$Hg(HS)_2$	0.17	0.18
	pH	**0.2**	**0.13**

Hg methylating group	Environmental factors	*r* (AB)	*P*
Desulfobulbus	MeHg/pH/tHg	**0.36**	**0.05**
Desulfobulbus	Sulfate	**0.41**	**0.06**
Desulfobulbus	$Hg(HS)_2$	**0.36**	**0.09**
Unclassified Deltaproteobacteria	MeHg	**0.53**	**0.11**
Unclassified Deltaproteobacteria	tHg	**0.35**	**0.06**
Unclassified Deltaproteobacteria	$Hg(HS)_2$	**0.44**	**0.08**
Geobacter	Sulfate	**0.37**	**0.13**
Geobacter	pH	**0.3**	**0.08**
Stenotrophomonas	pH/tHg/MeHg	**0.42**	**0.04**
Pseudomonas	MeHg	**0.15**	**0.02**

[a]Bray-Curtis dissimilarity matrix calculated from the taxa composition at phylum, class and genus level. [b]Euclidean distance matrix calculated from the environmental parameters (tHg, MeHg, sulfate, sulfide, $Hg(HS)_2$ and pH were included). Only significant parameters with *P* at 0.1 were considered for further analysis. [c]Bray-Curtis dissimilarity matrix calculated from the 9 mercury resistant taxa described in Table 7a. Only significant values are highlighted with bold fonts.

Supplementary Table S3. Oligonucleotides and barcode sequences used in the study.

Primer set	Individual primer	Sequence (5'-3')	Primer location	Annealing Temperature (^{0}C)
PCR, full-length SSU rRNA	27F<br>1492R	AGAGTTTGATCMTGGCTCAG<br>GGTTACCTTGTTACGACTT	8-27<br>1510-1492	55
ARISA, ITS	S-D-Bact-1522-b-S-20 (1522F)<br>L-D-Bact-132-A-A-18 (LSU132R)	TGCGGCTGGATCCCCTCCTT<br>CCGGGTTTCCCCATTCGG	1522–1541<br>132–115	50
Pyrosequencing, SSU rRNA	Gray28F<br>Gray519R	GAGTTTGATCNTGGCTCAG<br>GTNTTACNGCGGCKGCTG	9–27<br>536–519	60

Primers Gray28F and Gray519R extends across V1-V3 region of 16S rRNA gene.
